# Induction of ER stress-mediated apoptosis through SOD1 upregulation by deficiency of CHI3L1 inhibits lung metastasis

**DOI:** 10.7150/thno.82898

**Published:** 2023-04-29

**Authors:** Ji Eun Yu, In Jun Yeo, Seung Sik Yoo, Sung-hyun Kim, Dong Ju Son, Jaesuk Yun, Sang-Bae Han, Jin Tae Hong

**Affiliations:** College of Pharmacy and Medical Research Center, Chungbuk National University, 194-31, Osongsaengmyeong 1-ro, Osong-eup, Cheongju-si, Chungbuk 28160, Republic of Korea.

**Keywords:** Chitinas-3-like 1, ER stress, metastasis, superoxide dismutase-1

## Abstract

Chitinase-3-like protein 1 (CHI3L1), which is secreted by immune and inflammatory cells, is associated with several inflammatory diseases. However, the basic cellular pathophysiological functions of CHI3L1 are not well characterized. To investigate the novel pathophysiological function of CHI3L1, we performed LC-MS/MS analysis of cells transfected with Myc-vector and Myc-CHI3L1. We analyzed the changes in the protein distribution in Myc-CHI3L1 transfected-cells, and identified 451 differentially expressed proteins (DEPs) compared with Myc-vector-transfected-cells. The biological function of the 451 DEPs was analyzed and it was found that the proteins with endoplasmic reticulum (ER)-associated function were much more highly expressed in CHI3L1-overexpressing cells. We then compared and analyzed the effect of CHI3L1 on the ER chaperon levels in normal lung cells and cancer cells. We identified that CHI3L1 is localized in the ER. In normal cells, the depletion of CHI3L1 did not induce ER stress. However, the depletion of CHI3L1 induces ER stress and eventually activates the unfolded protein response, especially the activation of Protein kinase R-like endoplasmic reticulum kinase (PERK), which regulates protein synthesis in cancer cells. CHI3L1 may not affect ER stress owing to the lack of misfolded proteins in normal cells, but instead activate ER stress as a defense mechanism only in cancer cells. Under ER stress conditions induced by the application of thapsigargin, the depletion of CHI3L1 induces ER stress through the upregulation of PERK and PERK downstream factors (eIF2α and ATF4) in both normal and cancer cells. However, these signaling activations occur more often in cancer cells than in normal cells. The expression of Grp78 and PERK in the tissues of patients with lung cancer was higher compared with healthy tissues. It is well known that ER stress-mediated PERK-eIF2α-ATF4 signaling activation causes apoptotic cell death. ER stress-mediated apoptosis induced by the depletion of CHI3L1 occurs in cancer cells, but rarely occurs in normal cells. Consistent with results from the *in vitro* model, ER stress-mediated apoptosis was greatly increased during tumor growth and in the lung metastatic tissue of CHI3L1-knockout (KO) mice. The analysis of “big data” identified superoxide dismutase-1 (SOD1) as a novel target of CHI3L1 and interacted with CHI3L1. The depletion of CHI3L1 increased SOD1 expression, resulting in ER stress. Furthermore, the depletion of SOD1 reduced the expression of ER chaperones and ER-mediated apoptotic marker proteins, as well as apoptotic cell death induced by the depletion of CHI3L1 *in in vivo* and *in vitro* models. These results suggest that the depletion of CHI3L1 increases ER stress-mediated apoptotic cell death through SOD1 expression, and subsequently inhibits lung metastasis.

## Introduction

The endoplasmic reticulum (ER) is an important cellular organelle responsible for the synthesis of proteins, protein modification through glycosylation or disulfide bond formation, protein folding into a normal structure, calcium storage, and signaling transduction 1-4. Cells produce proteins to perform their own given functions, which are controlled selectively and precisely by environmental changes. Consequently, the “quality control system” for proteins in the ER must be precisely controlled and operated 5, 6. However, this process is perturbed by various situations, resulting in ER stress, which can cause problems with various cellular functions performed by ER. When ER stress occurs, the unfolded protein response (UPR) exerts a quality control action by increasing the expression of ER chaperones, suppressing the production of protein synthesis, and removing misfolded proteins when excess protein has accumulated in the ER.

The activation of UPR by ER stress is mediated by IRE-1 (inositol-requiring 1), PERK (Protein kinase R-like endoplasmic reticulum kinase), and ATF6 (activating transcription factor) 7-10. In the absence of ER stress, Grp78 (glucose-regulated protein of 78 kDa), and ER chaperone, inhibits its activity by binding to ATF6, PERK, and IRE-1. Under ER stress, Grp78 is dissociated from PERK, IRE1, and ATF6, and binds to unfolded proteins, activating the UPR. Although the UPR is naturally resolved by the cell signaling system in normal condition, but the excessive induction of ER stress cannot be resolved and causes various diseases. Many studies have reported that chronic ER stress is a feature of neurodegenerative diseases, cancer, metabolic diseases, and inflammatory diseases, and is related to the major pathogenesis of diseases 11-18. Therefore, studies on the regulation of UPR, a signaling mechanism of ER stress, have been increasing. Nevertheless, several aspects of the mechanism underlying the cellular response to ER stress and the UPR mechanism remain unknown.

Chitinase-3-llke protein 1 (CHI3L1, YKL-40) was first discovered in mouse breast cancer cells and was named breast regression protein 39 (BRP-39). CHI3L1 binds to chitin; however, unlike chitotriosidase (CHIT1) and acid mammalian chitinase (AMCase), it has an intrinsic function independent of chitin. In humans, CHI3L1 plays an important role in maintaining homeostasis in many organs 19-21. CHI3L1 is produced in monocytes, macrophages, neutrophils, cultured chondrocytes, and synovial cells, which are involved in cell proliferation and survival, and have mitogenic effects on skin, lung fibroblasts and synoviocytes 22-27. Similar to CHIT1, CHI3L1 exists within the human tissues of a healthy person and increases in several disease states. An increase in CHI3L1 is observed in malignant tumors, such as lung cancer, breast cancer, and melanoma, inflammation, rheumatoid arthritis, and osteoarthritis, as well as other conditions such as pyroclastic meningitis and community pneumonia 20, 25, 28-36. It is important to understand the precise biological function of CHI3L1 in the pathogenesis of cancer, allergic diseases, and inflammatory diseases. Although it is reported that CHI3L1 is localized to the ER, details are not known about its function 29, 37. In this study, we explored the novel pathophysiological function of ER CHI3L1 in the development of lung tumors.

## Methods

### Cell culture and treatment

A549 lung carcinoma, MRC-5 normal lung cell, U-2 OS bone osteosarcoma, A172 glioblastoma, Hep3B hepatocellular carcinoma, and B16-F10 *Mus musculus* skin melanoma cells were obtained from the American Type Culture Collection (Manassas, VA). U-2 OS cells were cultured in McCoy's 5a medium supplemented with 10% fetal bovine serum (FBS), 100 μg/mL penicillin, and 100 μg/mL streptomycin. A549, MRC-5, and B16F10 cells were cultured in RPMI 1640 medium supplemented with 10% FBS, 100 μg/mL penicillin, and 100 μg/mL streptomycin. A172 and Hep3B cells were cultured in DMEM medium supplemented with 10% FBS, 100 μg/mL penicillin, and 100 μg/mL streptomycin. Cell cultures were maintained in an incubator with a humidified atmosphere of 5% CO_2_ at 37 °C. Reduced serum medium and X-tream GENE HP DNA transfection reagent were used for transfecting plasmid and siRNA oligonucleotides following the manufacturer's instructions.

### Animal model

Animal experiments were performed as described previously 25. For lung metastasis model, A549 cells were injected into the tail veins of CHI3L1 KO mice and wild type mice (1 × 106 cells in /100 μl phosphate-buffered saline (PBS) per animal). 7 weeks after the injections, animals were sacrificed and the tumor lung metastases were counted on the lung surface. At the end of the experiment, the animals were sacrificed and the tumors were separated from the surrounding muscles and dermis, excised and weighed.

All protocols involving mice in this study were reviewed and approved by the Chungbuk National University Institutional Animal Care and Use Committee (IACUC) and complied with the Korean National Institute of Health Guide for the Care and Use of Laboratory Animals (CBNUA-1073-17-01).

### Western blot analysis

The tissues were homogenized with lysis buffer (20 mM Tris-HCl pH 7.8, 0.1% NP-40, 200 mM NaCl, 2 mM EDTA, 5 mM EGTA with protease inhibitor and protease inhibitor) and centrifuged at 13,000 X *g* for 20 min at 4 °C. Cells were harvested and lysed using lysis buffer and centrifuged. The proteins were loaded to SDS-PAGE. The specific primary antibodies and HRP-conjugated secondary antibodies were incubated and visualized by using a FUSION Solo S chemiluminescence detection system (Vilber Lourmat, Collégien, France).

### Immunoprecipitation

For immunoprecipitation, the cell lysates were incubated with specific primary antibodies for 4 h at 4 °C. Then protein A/G PLUS-Agrose (Santa cruz) were added to antibody containing cell lysates and incubated for 2 h at 4 °C. Immunoprecipitated proteins were loaded to SDS-PAGE.

### Immunocytochemistry

Cells were fixed with 4% paraformaldehyde in PBS for 15 min at room temperature. Cells then were permeabilized with 100% cold-Methanol for 5 min and blocked by 4% BSA in PBS with 0.1% Triton X-100 (PBST) for 1 h. Primary antibodies were incubated for overnight at 4 °C and Alexa Fluor 488 (#A32723, #A32731, Invitrogen) or Texas Red (#T-862, #T-2767, Invitrogen) conjugated secondary antibodies were incubated for 1h at room temperature. Fixed cells were incubated with 1 μg/mL of DAPI (#D9542, Sigma-Aldrich) for 5 min at room temperature and then covered with Fluoromount-G Mounting Medium (#0100-01, Southern Biotech, Birmingham, AL). Cells were visualized using Ziess AxioObserver (Carl Zeiss, Oberkochen, Germany) fluorescence microscope system. Digital images were analyzed using the ZEN 2.1 (Carl Zeiss) software.

### Immunohistochemistry (IHC)

The tumor and lung tissue sections were blocked with 3% normal goat serum diluted in PBS, for 30 min; the sections were then incubated with antibodies for CHI3L1, Grp78, PERK, CHOP, caspase 12, and SOD1, at the appropriate dilution in blocking serum, for overnight at 4 ºC. The slides were washed in PBS, followed by the avidin-biotin-peroxidase complex (#PK-6101, Vector Laboratories, Burlingame, CA). The slides were washed, and the peroxidase reaction was developed with diaminobenzidine and peroxide (#SK-4100, Vector Laboratories), mounted in Aqua-Mount, and evaluated under a light microscope (Olympus).

### TUNEL assay

A549 cells were transfected with either control siRNA or CHI3L1-siRNA for 1 day with thapsigargin for 18 h. Then cells were fixed with 4% paraformaldehyde in DPBS for 30 min at room temperature, permiabilized with 0.1% Triton X-100 in 0.1% sodium citrate for 10 min at room temperature. The cleavage DNA fragments were stained for 1 h in the dark at 37 °C using the TUNEL reaction mixture. Cells were observed on an Axio Observer Z1 inverted fluorescence microscope.

### Cell migration assay

For trans-well assay, A549 cells were seeded on upper chamber inserts (8.0 μm pore trans-well; Corning Inc., NY). After incubation for 18 h, the cells were fixed with 4% formaldehyde for 5 min, permeated with 100% methanol for 15 min, and stained with 0.1% crystal violet for 20 min. In the upper chamber, non-migrated cells were removed with a cotton swab. The migrated cells were visualized using a light microscope (Olympus) and analyzed using ImageJ software (NIH).

### Cell lysis and protein digestion

Cell pellets were stored at -80 °C until cell lysis was performed. Lysis of cell pellets was done at room temperature. Biological replicates (one cell pellet from one cell line) were processed simultaneously to minimize the effect of error. Pellets were resuspended in 100 μL 8 M Urea (Merck, Branchburg, NJ) were added. Protein concentration was estimated with the bicinchoninic acid assay (Pierce, Rockford, IL). Proteins were reduced with 10 mM dithiothreitol (Sigma) and alkylated 25 mM iodoacetamide (Sigma). Samples were diluted in 50 mM AmBic and trypsinized (Promega, Madison, WI) overnight at 37 °C at a trypsin/protein ratio of 1:50, w/w). The resulting peptide mixture was lyophilized overnight and digested peptides were cleaned by flowing through an Oasis HLB 1cc (10 mg) solid phase extraction (SPE) catridges (Oasis, Milford, MA). Samples were dried using a Speed-Vac (Thermo Savant, Holbrook, NY) and stored at -80 °C until time for analysis. The peptides digestion protocols were performed modifying the method described previously 38.

### LC-MS/MS for global proteomics

LC-MS/MS was performed as described previously 39. Nano-high-performance liquid chromatography (nano-LC) analyses were performed using an Easy n-LC 1000 system (Thermo Fisher Scientific, San Jose, CA). The column (15 cm x 75 μm) was packed in-house with Jupiter 2 μm, 100 Å pore size C18 beads (Phenomenex, Torrance, CA). Mobile phase A for LC separation consisted of 0.1% formic acid in deionized water and the mobile phase B consisted of 0.1% formic acid in acetonitrile. For analysis of fractionated samples, the mobile phase was programmed from 5% B over 10 min, 5% B to 30% B over 35 min, 30% B to 90% B over 12 min, and finally to 90% B to 5% B over 13 min at a flow rate of 300 nL/min. A Q-ExactiveTM mass spectrometer (Thermo Fisher) was used for MS analyses and was operated with Xcalibur (version 2.1, Thermo Fisher) to generate peak lists. For peptide ionization, 2400 V was applied and a 250 °C capillary temperature was used. The full scan event was collected using a m/z 350 - 2000 mass selection, an Q-Exactive MS resolution of 70,000, a target automatic gain control (AGC) value of 1 X 10^6^, and a maximum injection time of 80 ms. Fragmentation was performed with a normalized collision energy of 25.

### Human samples

Human tissue samples from lung cancer patients and normal controls were obtained from Chungbuk National University Hospital Biobank, Keimyung University Dongsan Hospital Biobank and the Biobank of Ajou University Hospital, members of Korea Biobank Network. All studies using human samples were conducted in accordance with the Declaration of Helsinki and were approved by the Ethics Committee of Chungbuk National University Medical Center (IRB No. CBNU-201910-BR-941-01).

### Statistical analysis

Statistical analyses were performed using the GraphPad Prism 5 software. All error bars reported are the standard deviation (SD) unless otherwise indicated. Pairwise comparisons were performed using Student's *t*-test. Multiple comparisons were using one-way analysis of variance followed by Tukey's tests. Differences between groups were considered significant at *P*-values of <0.05.

## Results

### Proteomics analysis of association of CHI3L1 with ER

**Figure 1:**
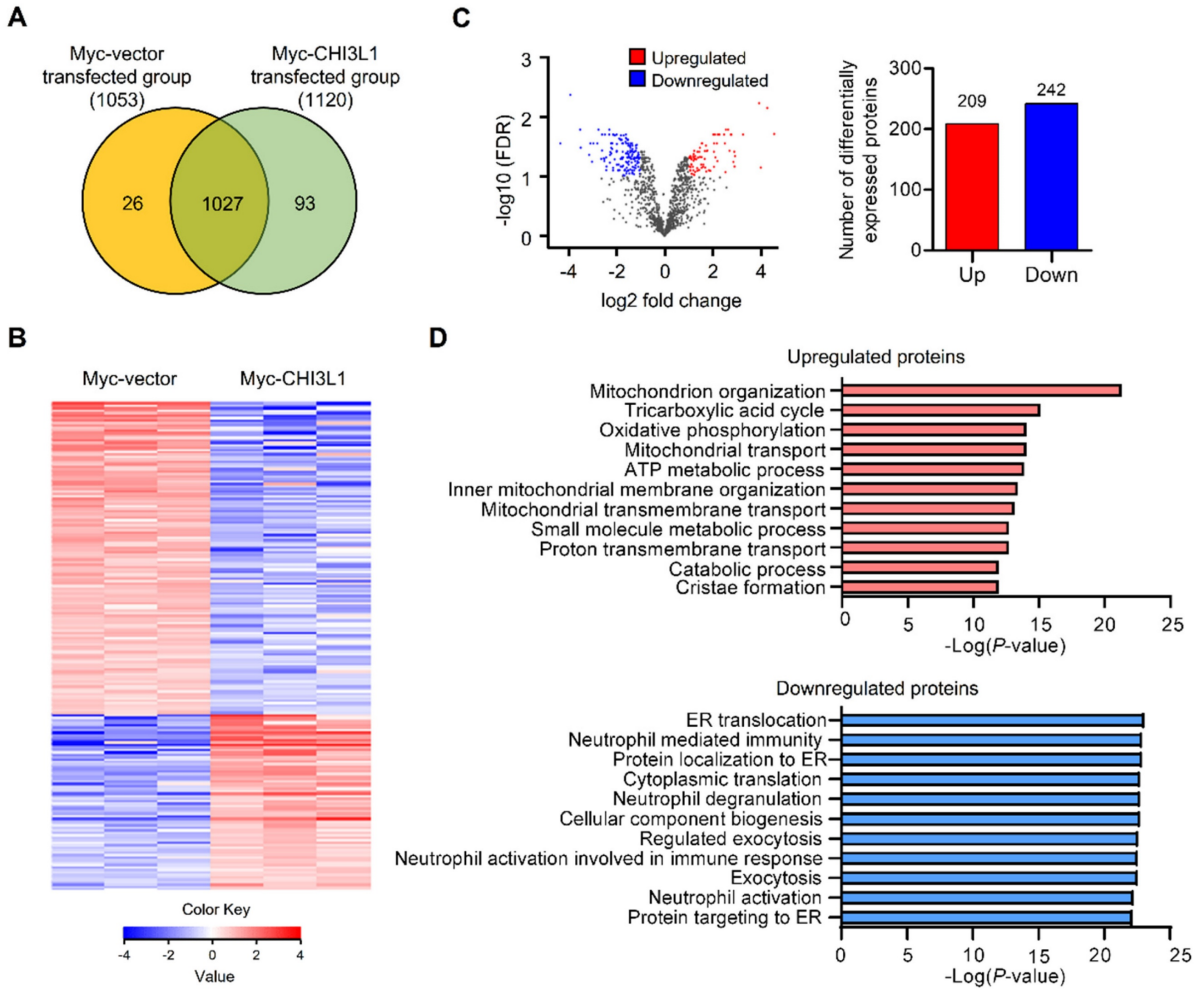
** ER associated proteins are the most enriched in the downregulated DEPs in CHI3L1 expressing cells.** (**A**) Venn diagram of identified differential expressed proteins (DEPs) from LC-MS/MS. 1027 DEPs are shared between both groups (1053 for the Myc-vector and 1120 for the Myc-CHI3L1 transfected cells). (**B**) Heatmap of expression profiles for differential proteins. The color scale represents average log signal intensity values. Red indicated upregulated protein, and blue indicated the downregulated protein in comparison groups. (**C**) Volcano plot of DEPs between Myc-vector and Myc-CHI3L1 transfected cells. Red dots indicate upregulated DEPs (log_2_FC > 1), blue dots indicate downregulated DEPs (log_2_FC < -1), and gray dots indicate no significant difference between Myc-vector and Myc-CHI3L1 transfected cells. FDR < 0.05; *p* < 0.05. In total 451 DEPs, including 209 upregulated and 242 downregulated DEPs, were identified. (**D**) The gene ontology categories of DEPs in biological process. Red bar graph indicates upregulated DEPs in Myc-CHI3L1 transfected cells compared with control. The top 10 significant Biological Processes terms of upregulated DEPs in Myc-CHI3L1 transfected cells. The enrichment p value in biological processes is indicated.

To evaluate the global changes in protein abundance in CHI3L1-expressing cells, we performed nano-high-performance liquid chromatography (nano-LC). In total, we identified 1146 proteins and 1120 proteins in CHI3L1-expressing cells and 1053 proteins in control vector-expressing cells. There were 1027 proteins differentially expressed in CHI3L1-expressing cell and vector-expressing cell samples (Figure 1A and 1B). Using a volcano plot, we identified 451 differentially expressed proteins (DEPs) in CHI3L1-expressing cells, which comprised 209 upregulated proteins and 242 downregulated proteins compared with vector-expressing cells (fold change > 2 and P value <0.05) (Figure 1C). To identify the functional relationships, functional annotation was performed by Gene Ontology (GO) analysis for the upregulated and downregulated DEPs. With regard to GO biological process terms, significant enrichment was found in “mitochondrion organization”, “tricarboxylic acid cycle”, and “oxidative phosphorylation” for the top three upregulated DEPs and in “ER translocation”, “neutrophil-mediated immunity”, and “protein localization to ER” for the top three downregulated DEPs (Figure 1D). Further analysis showed that the most significant change was “ER translocation”, and the ER associated proteins, specifically in the downregulated DEPs in the CHI3L1-expressing cells.

### CHI3L1 is abundant in the ER

**Figure 2:**
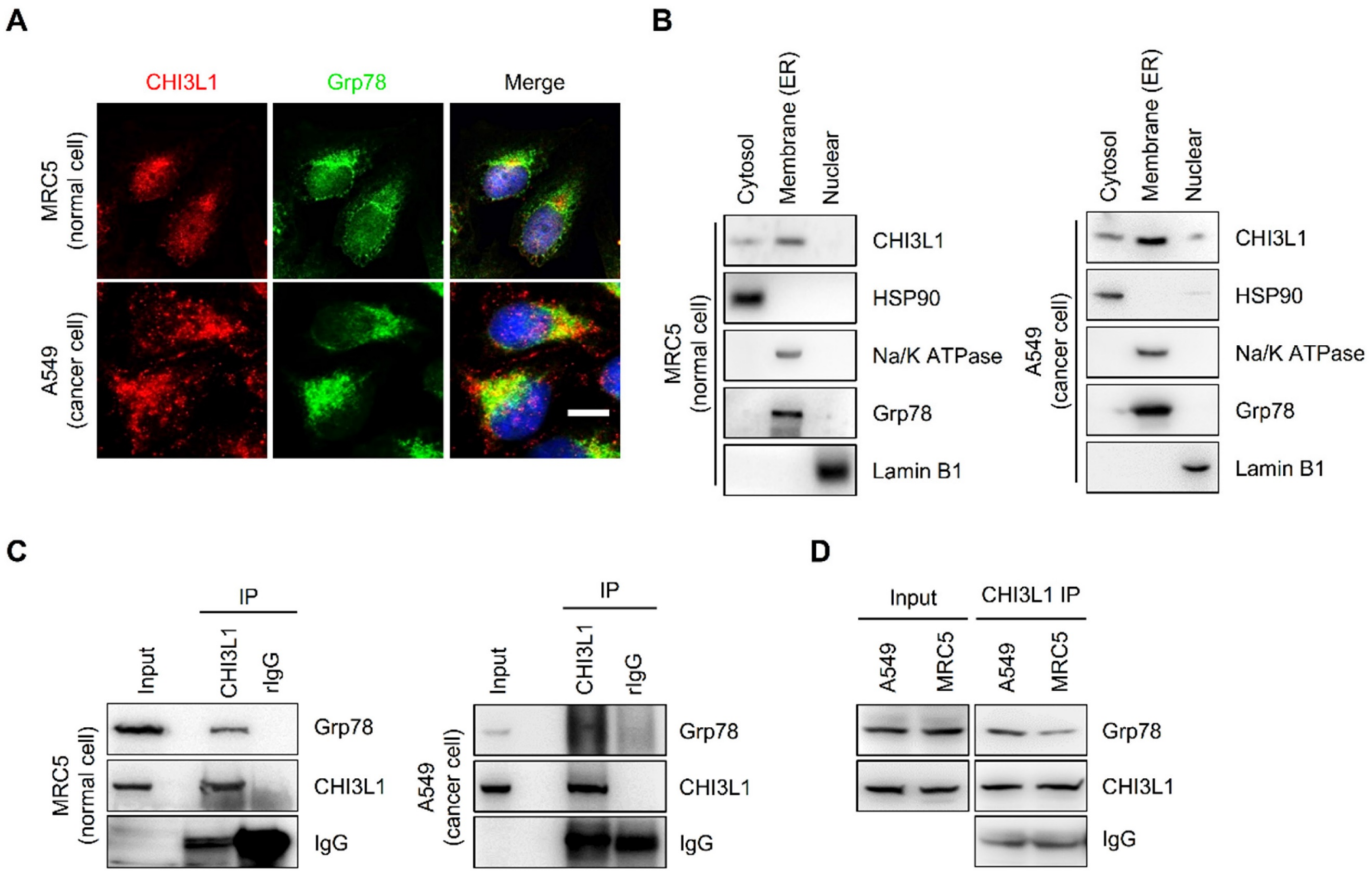
** CHI3L1 is localized in ER and associated with ER chaperone Grp78.** (**A**) The 4% paraformaldehyde fixed MRC5 (normal lung cell) and A549 (lung cancer cell) cells were co-stained with anti-CHI3L1 and anit-Grp78 (endoplasmic reticulum marker). Scale bar, 10 μm. (**B**) MRC5 and A549 cel0ls were fractioned into three distinct fractions, including cytosolic, membrane bound organelle, and nuclear fractions. Isolated proteins were subjected to immunoblot analysis with indicated antibodies. (**C, D**) MRC5 and A549 cell lysates were immunoprecipitated using anti-CHI3L1 antibody, and then subjected to immunoblot analysis with the indicated antibodies.

To identify the potential association between ER and CHI3L1, the subcellular localization of CHI3L1 was confirmed using immunocytochemistry. CHI3L1 was localized to overall cytoplasm and, is particularly enriched in the ER in MRC5 normal lung cells (Figure 2A). In addition, in various cancer cell lines, including A549 lung cancer cells, A172 glioblastoma cells, U-2 OS osteosarcoma cells, and Hep3B liver cancer cells, a novel localization of CHI3L1 was identified (Figure 2A and Supplemental Figure S1A). The results revealed that CHI3L1 was abundantly localized to the ER. Furthermore, the analysis of the fractionation assay revealed that CHI3L1 was expressed in the ER/golgi membranes, the nuclear, and the cytosol in both normal and cancerous lung cells (Figure 2B). To determine the interaction between CHI3L1 and ER-associated protein, we performed an immunoprecipitation assay. The results showed that endogenous CHI3L1 and Grp78 interacted in both normal and cancerous lung cells (Figure 2C); however, the interaction between CHI3L1 and Grp78 was greater in lung cancer cells compared with normal lung cells (Figure 2D). These results suggest that CHI3L1 is enriched in the ER and may be associated with lung cancer development.

### CHI3L1 regulates the expression of ER chaperone proteins

**Figure 3:**
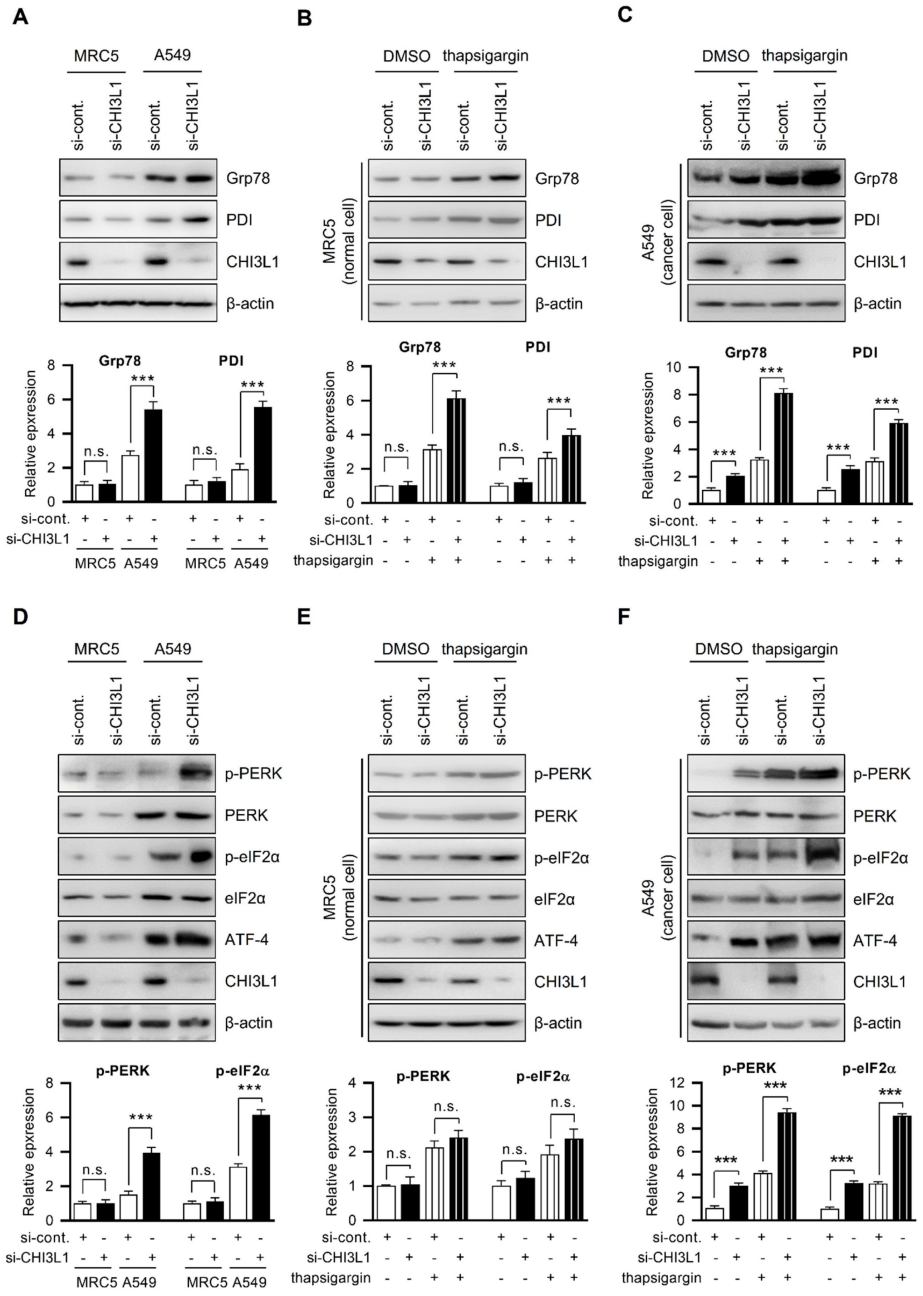
** Depletion of CHI3L1 activates ER stress and induces PERK and eIF2α in A549 cells.** (**A**) MRC5 (normal lung cell) and A549 (lung cancer cell) cells were transfected with control siRNA (si-cont.) or CHI3L1 siRNA (si-CHI3L1). The cell lysates were subjected to immunoblot analysis with the indicated antibodies. The intensity of each band was measured and the ratio of the amount of each protein to β-actin was calculated. Data are presented as mean ± standard deviation (SD) from two independent experiments. ***, *P* < 0.001; n.s., not significant. (**B**) MRC5 cells were transfected with si-control or si-CHI3L1, then treated with 1 uM thapsigargin (ER stress inducer) for 18 h. The cell lysates were subjected to immunoblot analysis with the indicated antibodies. The intensity of each band was measured and the ratio of the amount of each protein to β-actin was calculated. Data are presented as mean ± standard deviation (SD) from two independent experiments. ***, *P* < 0.001; n.s., not significant. (**C**) A549 cells were transfected with si-control or si-CHI3L1, then treated with 1 uM thapsigargin for 18 h. The cell lysates were subjected to immunoblot analysis with the indicated antibodies. The intensity of each band was measured and the ratio of the amount of each protein to β-actin was calculated. Data are presented as mean ± standard deviation (SD) from two independent experiments. ***, *P* < 0.001. (**D**) MRC5 (normal lung cell) and A549 (lung cancer cell) cells were transfected with control siRNA (si-cont.) or CHI3L1 siRNA (si-CHI3L1). The cell lysates were subjected to immunoblot analysis with the indicated antibodies. The intensity of each band was measured and the ratio of the amount of each protein to β-actin was calculated. Data are presented as mean ± standard deviation (SD) from two independent experiments. n.s., not significant. (**E**) MRC5 cells were transfected with si-control or si-CHI3L1, then treated with 1 uM thapsigargin (ER stress inducer) for 18 h. The cell lysates were subjected to immunoblot analysis with the indicated antibodies. (**F**) A549 cells were transfected with si-control or si-CHI3L1, then treated with 1 uM thapsigargin for 18 h. The cell lysates were subjected to immunoblot analysis with the indicated antibodies. The intensity of each band was measured and the ratio of the amount of each protein to β-actin was calculated. Data are presented as mean ± standard deviation (SD) from two independent experiments. ***, *P* < 0.001.

The ER is critical for protein homeostasis, and many studies have found that ER stress occurs in a variety of diseases, including cancers, and is linked to the pathogenesis of disease 40-43. We investigated how CHI3L1 causes ER stress in normal cells and cancer cells. Thus, we first compared ER chaperone protein (Grp78 and PDI) levels in MRC5 cells (normal lung cells) and A549 cell (lung cancer cells). The results showed that ER chaperone protein levels were higher in A549 cells than in MRC5 cells (Figure 3A). Next, we examined whether CHI3L1 influenced the levels of ER chaperone proteins. CHI3L1 did not affect the ER chaperone protein levels such as Grp78 and PDI in MRC5 cells (Figure 3A). However, in contrast to normal cells, the depletion of CHI3L1 increased the expression of ER chaperone proteins in A549 cells (Figure 3A). Then, we investigated whether CHI3L1 affected the expression of ER chaperone proteins during ER stress. The expression of ER chaperone proteins was increased by the application of thapsigargin, a compound known to induce ER stress in normal cells. Unlike DMSO treatment, the depletion of CHI3L1 under ER stress conditions increased the expression of ER chaperone proteins in normal cells (Figure 3B). In addition, we analyzed the mRNA level of ER chaperon proteins by RT-qPCR. The results showed that depletion of CHI3L1 did not change the mRNA level of ER chaperon proteins in MRC5 normal cells. However, the depletion of CHI3L1 increased the mRNA level of ER chaperone proteins in thapsigargin treated MRC5 normal cells (Supplementary Figure S2A). In A549 cells treated with thapsigargin alone, ER chaperone protein levels were also increased compared with control treated cancer cells (Figure 3C). In addition, the depletion of CHI3L1 significantly increased the expression of ER chaperone proteins in ER stress conditions in lung cancer cells (Figure 3C). Similar to protein levels, the depletion of CHI3L1, as well as thapsigargin treatment, increased the mRNA expression level of ER chaperone proteins (Supplementary Figure S2B).

To confirm whether CHI3L1 induces ER stress in lung cancer cells, ER chaperone protein levels were examined by 4-phenylbutyric acid (4-PBA) treatment, an ER stress inhibitor. The western blotting results showed that 4-PBA treatment had no obvious effect on ER chaperone protein levels in cells transfected with control siRNA. However, ER chaperone protein levels were lower in CHI3L1 depletion in the presence of 4-PBA, indicating that the degree of ER stress is reduced (Supplemental Figure S2C). These results indicated that the depletion of CHI3L1 induces ER stress in lung cancer cells. In ER stress conditions, the depletion of CHI3L1 increased the ER chaperone protein levels in both normal and cancerous lung cells. However, the ER chaperone protein levels were significantly higher in cancer cells than in normal cells.

### Depletion of CHI3L1 induces the PERK-eIF2α pathway

The ER regulates a variety of cellular functions, including correct protein folding, protein synthesis, and calcium homeostasis 1-4. Numerous stimuli under physiological or pathological circumstances cause the accumulation of unfolded proteins in the ER, which then activates unfolded protein response (UPR) and, if the stimulus is severe or prolonged, leads to cell death 11-18. PERK and UPR sensor proteins are phosphorylated under ER stress and inhibit protein translation through eIF2α phosphorylation. This process contributes to improvement in the protein folding ability of ER by allowing the protein translation of mRNA encoding transcription factor ATF-4 41, 44-46. We investigated the PERK signal transduction pathways changed by CHI3L1. First, we confirmed the expression of PERK and PERK downstream factors (eIF2α phosphorylation and ATF-4) in MRC5 cells and A549 cells. Compared with normal cells, PERK phosphorylation, eIF2α phosphorylation, and ATF-4 levels were higher in A549 lung cancer cells (Figure 3D). Next, we investigated whether the depletion of CHI3L1 affects the expression of PERK and its downstream factors. CHI3L1 did not affect PERK phosphorylation, eIF2α phosphorylation, or ATF-4 levels in MRC5 cells (Figure 3D). However, the depletion of CHI3L1 further increased PERK phosphorylation, eIF2α phosphorylation, and ATF-4 levels in A549 cells (Figure 3D). We then investigated the expression of PERK signaling factors (PERK, eIF2α, and ATF-4) in the depletion of CHI3L1 cells subjected to ER stress. The induction of ER stress by thapsigargin treatment in normal cells increased PERK phosphorylation, eIF2α phosphorylation and ATF-4 levels (Figure 3E).

When normal cells were subjected to ER stress, the depletion of CHI3L1 caused a slight further increase in PERK singling protein levels (Figure 3E). Depletion of CHI3L1 did not significantly affect the mRNA level of *ATF-4*. However, when these cells were treated with thapsigargin, the depletion of CHI3L1 increased the mRNA level of *ATF-4* (Supplementary Figure S2D). The PERK signaling protein levels were increased by thapsigargin treatment in lung cancer cells (Figure 3F). Under ER stress-induced by thapsigargin treatment, the depletion of CHI3L1 greatly increased PERK protein levels in lung cancer cells (Figure 3F). In addition, we observed an increase in the mRNA expression of *ATF-4* following thapsigargin treatment and CHI3L1 depletion, which further supports the findings of increased protein levels (Supplementary Figure S2E).

Finally, we assumed that CHI3L1 depletion-induced ER stress would eventually activate PERK signaling. To confirm the PERK signaling protein levels, A549 cells were treated with the ER stress inhibitor 4-PBA. The levels of PERK signaling protein were reduced by 4-PBA treatment. 4-PBA inhibits the increase in PERK protein levels induced by the depletion of CHI3L1 (Supplementary Figure S2F). Together, our data suggest that the depletion of CHI3L1 induces ER stress and UPR activation in lung cancer cells. Under stress conditions, the depletion of CHI3L1 greatly induces ER stress through the modulation of PERK-eIF2α-ATF4 signaling activation.

### Depletion of CHI3L1 induces ER stress-associated apoptosis

Several studies have reported that UPR activation by ER stress induces apoptosis 44, 47-50. PERK-eIF2α-ATF4 signaling activation can cause ER stress-mediated apoptosis 41, 44-46. We therefore evaluated the effect of CHI3L1 on ER stress-induced apoptosis. To verify whether CHI3L1 induces apoptosis via UPR processes, siRNA targeting CHI3L1 and control siRNA were transfected into MRC5 cells. After treatment with thapsigargin, the effect of CHI3L1 on cell proliferation was evaluated using the MTT assay. The depletion of CHI3L1 decreased the proliferation of MRC5 cells. Compared with the control, thapsigargin treatment decreased MRC5 cell proliferation. Furthermore, proliferation of cells treated with CHI3L1 siRNA after thapsigargin treatment was further decreased (Supplemental Figure S3A). Next, we investigated the apoptotic effect of CHI3L1 using the TUNEL assay. The TUNEL assay results showed that the depletion of CHI3L1 increased the proportion of apoptotic cells in MRC5 cells (1.39±0.40% vs 4.99±1.17%). Thapsigargin treatment also induced apoptosis (4.71±1.80%); however, thapsigargin treatment accelerated the effect of CHI3L1 siRNA on apoptotic cells (19.81±3.14%) (Supplemental Figure S3B). To further verify how the depletion of CHI3L1 affects apoptosis under ER stress, immunoblotting assays were performed. CHOP activation and caspase-12 cleavage is typical in apoptosis where ER stress is activated 44, 49, 51-53. Our results showed that the depletion of CHI3L1 did not change CHOP and cleaved caspase-12 levels in MRC5 cells. However, the depletion of CHI3L1 slightly increased the Bax/Bcl2 ratio in normal cells. Thapsigargin treatment slightly increased the ER stress-induced protein levels (CHOP and caspase 12) and the Bax/Bcl2 ratio. Following the induction of ER stress by thapsigargin, the depletion of CHI3L1-mediated apoptosis was significantly increased (Supplemental Figure S3C). In addition, we confirmed that the depletion of CHI3L1 affects cell migration during ER stress. The depletion of CHI3L1 slightly reduced MRC5 cell migration. Thapsigargin treated depletion of CHI3L1 cells significantly decreased normal cell migration (Supplemental Figure S3D). These results indicated that the depletion of CHI3L1 has no effect on ER-mediated apoptosis in normal cells. However, under ER stress, the depletion of CHI3L1 induces ER stress-mediated apoptosis in MRC5 cells.

**Figure 4:**
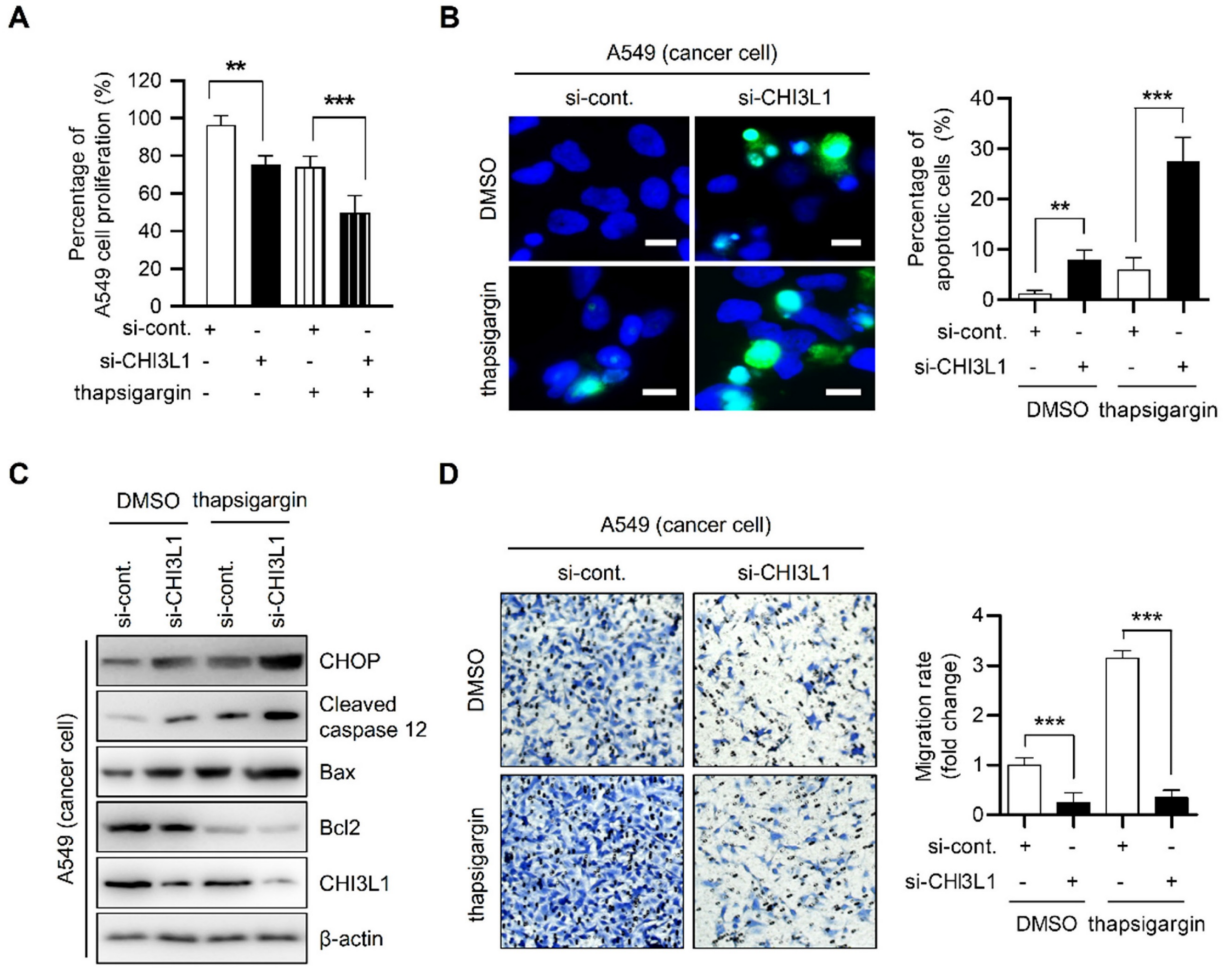
** Depletion of CHI3L1 induces ER stress mediated apoptosis via PERK- eIF2α-CHOP pathway in A549 cells.** (**A**) A549 cells were transfected with si-control or si-CHI3L1, then treated with 1 uM thapsigargin (ER stress inducer) for 18 h. The cell viability was measured using the MTT assay. The data are presented as the mean ± SD of three independent experiments. **, *P* < 0.01; ***, *P* < 0.001. (**B**) Representative fluorescence microscopic images showing DAPI (blue) and TUNEL (green) staining in A549 cells transfected with si-control or si-CHI3L1 in the absence or presence of the thapsigargin (1 μM). Scale bar, 10 μm. The number of positively stained cells was counted in three different fields and averaged. The data are presented as the mean ± SD of three independent experiments. **, *P* < 0.01; ***, *P* < 0.001. (**C**) A549 cells were transfected with si-control or si-CHI3L1, then treated with 1 uM thapsigargin for 18 h. The cell lysates were subjected to immunoblot analysis with the indicated antibodies. (**D**) si-control or si-CHI3L1 transfected A549 cells were seeded in the upper chamber and incubated at 37 °C for 18 h. The migrated cells on the bottom chamber were stained with 0.1% crystal violet. Data are presented as mean ±SD from three independent experiments. Scale bar, 100 μm. The number of migrated cells was counted in three different fields and averaged. The data are presented as the mean ± SD of three independent experiments. ***, *P* < 0.05.

To verify the effect of CHI3L1 depletion on ER stress-mediated cancer cell death in lung cancer cells, we performed MTT assay. The depletion of CHI3L1 moderately decreased the proliferation of A549 cells (96.35±4.42% vs 74.01±5.00%). Compared with DMSO-treated control cells, thapsigargin treatment did not alter cell proliferation (75.34±4.07%). The proliferation of cancer cells transfected with CHI3L1 siRNA and treated with thapsigargin was significantly decreased (49.88±7.80%) (Figure 4A). Next, apoptosis of cancer cells was detected using the TUNEL assay. Compared with control siRNA transfected-cells, apoptotic cancer cells were moderately increased in CHI3L1-depleted cells (1.31±0.52 vs 6.07±2.10%) (Figure 4B). In contrast, in normal cells, thapsigargin treatment increased the proportion of apoptotic cancer cells (7.96±1.77%). In addition, apoptosis in cells transfected with CHI3L1 siRNA and treated with thapsigargin was significantly increased in cancer cells (27.50±4.37%). Next, we investigated the levels of ER stress-induced marker proteins of apoptosis (CHOP and caspase 12) levels in cancer cells. The depletion of CHI3L1 increased the CHOP, cleaved caspase 12 levels and Bax/Bcl2 ratio in A549 lung cancer cells. Thapsigargin treated-cancer cells also increased the ER stress-induced apoptotic marker protein levels and Bax/Bcl2 ratio. In the treatment of thapsigargin, the depletion of CHI3L1 significantly increased the expression of ER stress-induced apoptotic marker proteins (Figure 4C). In agreement with the results of apoptotic cell death, the depletion of CHI3L1, as well as thapsigargin treatment, reduced A549 cell migration. Under thapsigargin induced stress, the depletion of CHI3L1 greatly eliminated cancer cell migration (Figure 4D). These results indicated that the depletion of CHI3L1 significantly increased the ER stress-mediated apoptotic cell death in lung cancer cells.

To confirm whether ER stress has an important role in apoptosis induced by the depletion of CHI3L1, A549 cells were pretreated with 4-PBA for 1 h and then transfected with CHI3L1 siRNA. Cell proliferation did not change in control siRNA treated with 4-PBA. However, 4-PBA prevented the reduction of cancer cell proliferation by the depletion of CHI3L1 (Supplemental Figure S4A). As shown by the results of the TUNEL assay, 4-PBA treatment did not affect cancer cell apoptosis in control siRNA. However, apoptosis induced by the depletion of CHI3L1 was significantly prevented by 4-PBA treatment (Supplemental Figure S4B). Similarly, immunoblotting results showed that 4-PBA treatment had no effect on apoptosis marker proteins. However, 4-PBA treatment decreased the ER stress-induced apoptosis marker protein levels, which were increased by the depletion of CHI3L1 (Supplemental Figure S4C). In the analysis of cancer cell migration using a Transwell assay, the treatment of 4-PBA has no effect on cancer cell migration. The application of 4-PBA resulted in similar cell migration to the control and in the depletion of CHI3L1 (Supplemental Figure S4D).

### CHI3L1 KO suppresses tumorigenesis in mice through ER-mediated apoptosis

**Figure 5:**
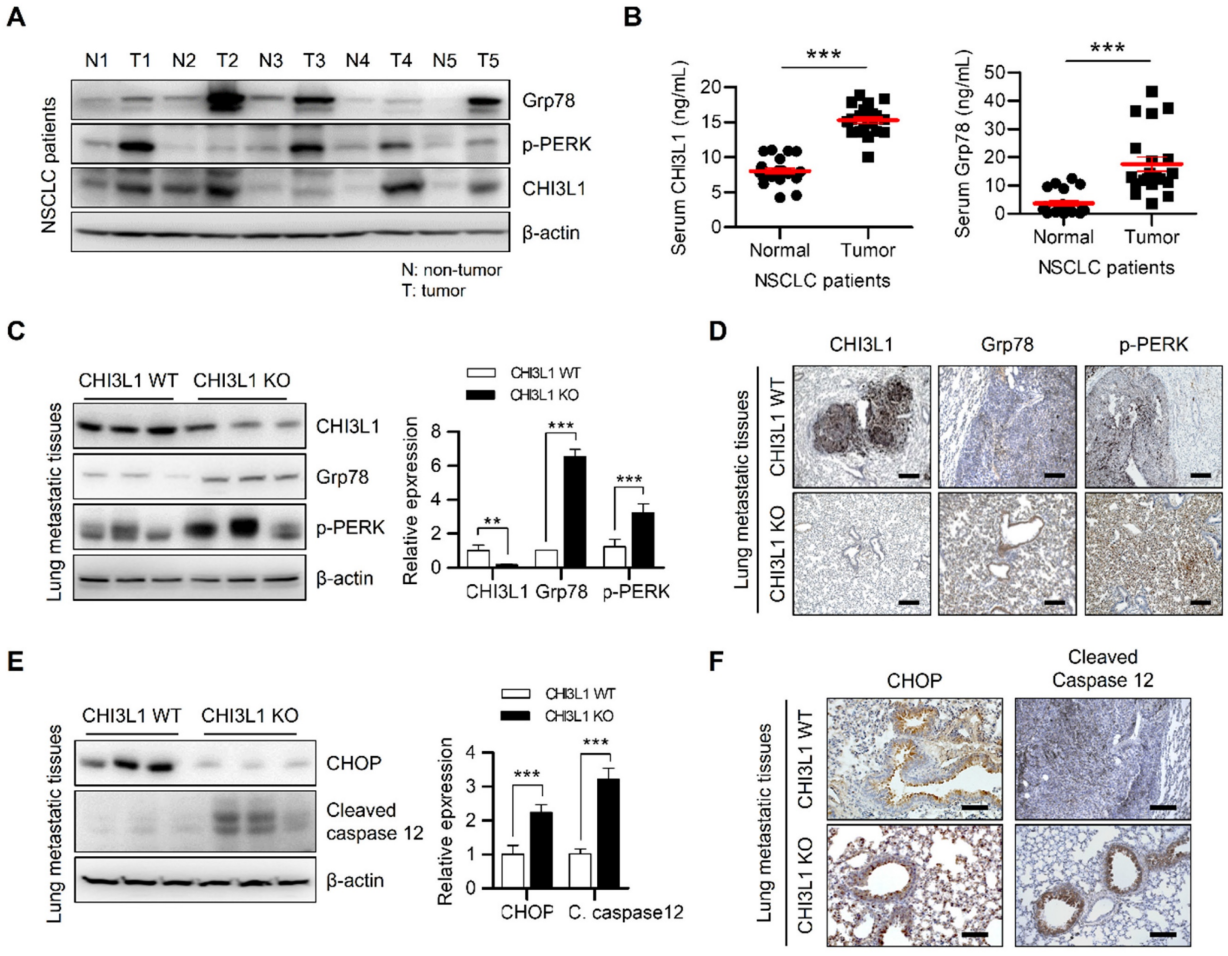
** The metastatic lung tissues of CHI3L1 KO mice induces ER stress mediated apoptosis.** (**A**) The lung tissue extracts of NSCLC patients were subjected to immunoblot analysis with indicated antibodies. (**B**) The serums of NSCLC patients were subjected to ELISA analysis with indicated proteins. (**C**) The metastatic lung tissue extracts of mice were subjected to immunoblot analysis with indicated antibodies. The intensity of each band was measured and the ratio of the amount of each protein to β-actin was calculated. Data are presented as mean ± standard deviation (SD) from two independent experiments. ***, *P* < 0.001. (**D**) Representative immunohistochemical images of metastatic lung tissues in mice using anti-CHI3L1, anti-Grp78, and anti-p-PERK antibodies in each group. Scale bar, 100 μm. (**E**) The metastatic lung tissues extracts of mice were subjected to immunoblot analysis with indicated antibodies. The intensity of each band was measured and the ratio of the amount of each protein to β-actin was calculated. Data are presented as mean ± SD from two independent experiments. ***, *P* < 0.001. (**F**) Representative immunohistochemical images of metastatic lung tissues in mice using anti-CHOP and anti-cleaved caspase12 antibodies in each group. Scale bar, 100 μm.

The activation of multiple ER stresses promotes tumor formation and metastasis in malignant cells 44, 51, 52. We subsequently investigated the expression of UPR-associated proteins in patients with lung cancer. The expression of Grp78 and PERK was increased in tissues from patients with lung cancer (Figure 5A). In conjunction with these changes in expression, CHI3L1 was also highly expressed in lung tumor tissue from patients with cancer (Figure 5A and 5B). Several studies have reported that deficiency of CHI3L1 suppresses lung tumor growth and metastasis 20, 25, 28-36. We therefore determined whether the effect of CHI3L1 on the UPR influenced lung metastasis. First, we confirmed the representative UPR-associated protein (Grp78 and p-PERK) levels in healthy lung tissue. The results showed no difference in UPR-associated protein levels in the normal lung tissues of CHI3L1 and KO mice (Supplementary Figure S5A). However, as shown in the Figure 5C, the protein levels of Grp78 and phosphorylated PERK were increased in the metastatic lung tumor tissues of CHI3L1 KO mice. Furthermore, the immunohistochemistry analysis results showed that the expression of Grp78 and phosphorylated PERK was significantly increased in metastatic lung tissues of CHI3L1 KO mice (Figure 5D), which is similar to the *in vitro* results. To verify whether the suppression of lung metastasis in CHI3L1 KO mice occurred via the UPR system, we performed western blotting assay. The expression of Grp78 and phosphorylated PERK was also increased in tumor tissues from CHI3L1 KO mice (Supplemental Figure S5B). These results were confirmed once again using immunohistochemistry staining. Immunohistochemistry analysis showed increased expression of Grp78 and phosphorylated PERK in tumor tissues of CHI3L1 KO mice (Supplemental Figure S5C). These results showed that ER stress is derived in CHI3L1 KO mice through the PERK signaling pathway in lung tumorigenesis.

Next, we investigated whether the inhibition of lung tumorigenesis in CHI3L1 KO mice was generated thorough ER stress-mediated apoptosis. Western blotting analyses of the metastatic lung tumor tissues from CHI3L1 KO mice significantly increased the level of CHOP and cleaved caspase-12, which are both ER stress-mediated apoptotic marker proteins (Figure 5E). Consistent with immunoblotting results, the immunohistochemical analysis also showed increased CHOP and cleaved caspase 12 levels in tumor tissues from CHI3L1 KO mice (Figure 5F). In addition, in the xenograft model, ER chaperone protein levels were significantly increased in tumor tissues from CHI3L1 KO mice (Supplementary Figure S5D and S5E). These results showed that CHI3L1 KO causes ER stress-mediated apoptosis in mice, and thus suppresses lung tumorigenesis.

### CHI3L1 regulates superoxide dismutase-1 expression

**Figure 6:**
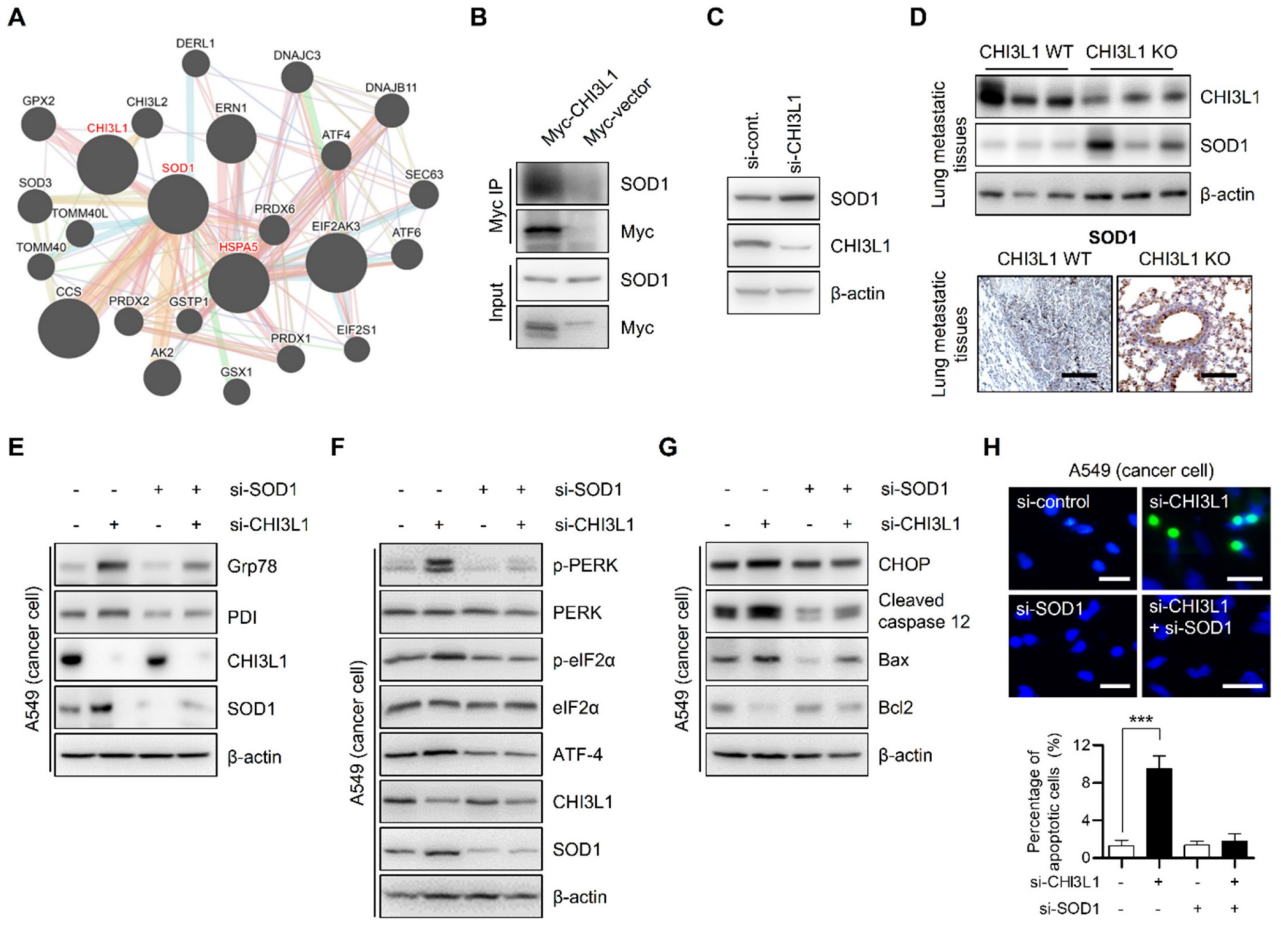
** SOD1 is involved in the depletion of CHI3L1-induced ER stress.** (**A**) A549 cells were transfected with either Myc-vector or Myc tagged Chi3L1 plasmids. The cell lysates were immunoprecipitated using the anti-Myc antibody, and then subjected to immunoblot analysis with the indicated antibodies. (**B**) A549 cells were transfected with either Myc-vector or Myc tagged Chi3L1 plasmids. The cell lysates were immunoprecipitated using the anti-Myc antibody, and then subjected to immunoblot analysis with the indicated antibodies. (**C**) A549 cells were transfected with control siRNA or si-CHI3L1.The cell lysates were subjected to immunoblot analysis with the indicated antibodies (**D**) The metastatic lung tissues extracts were subjected to immunoblot analysis with anti-SOD1 antibodies (upper). Representative immunohistochemical images of metastatic lung tissues using anti-SOD1 antibodies in each group (lower). Scale bar, 100 μm. (**E**) A549 cells were transfected with si-control, si-CHI3L1, or si-SOD1. The cell lysates were subjected to immunoblot analysis with the indicated antibodies. (**F**) A549 cells were transfected with si-control, si-CHI3L1, or si-SOD1. The cell lysates were subjected to immunoblot analysis with the indicated antibodies. (**G**) A549 cells were transfected with si-control, si-CHI3L1, or si-SOD1. The cell lysates were subjected to immunoblot analysis with the indicated antibodies. (**H**) Representative fluorescence microscopic images showing DAPI (blue) and TUNEL (green) staining in A549 cells transfected with si--control, si-CHI3L1, or si-SOD1. Scale bar, 20 μm. The number of positively stained cells was counted in three different fields and averaged. The data are presented as the mean ± SD of three independent experiments. ***, *P* < 0.001.

Next, we sought to investigate the detailed mechanism for the regulation of ER stress-mediated apoptosis by CHI3L1. We used the GENEMANIA online database to identify the proteins interacting with CHI3L1 and ER associated proteins. The GENEMANIA database showed that CHI3L1 is associated with superoxide dismutase-1 (SOD1) (Figure 6A). In several papers, the accumulation of SOD1 has been reported as one of the indicators of ER stress 54-56. To confirm whether CHI3L1 interacts with SOD1, we performed immunoprecipitation analysis. Figure 6B shows that CHI3L1 interacts with SOD1. Next, we investigated whether CHI3L1 affected SOD1 protein levels to regulate ER stress. The results showed that CHI3L1 did not affect SOD1 levels in MRC5 cells (Supplementary Figure S6A). However, unlike normal cells, the depletion of CHI3L1 caused dysregulation of the protein levels of SOD1 in A549 cells (Figure 6C). These results were confirmed once again in an *in vivo* model. There was slightly higher expression of SOD1 in healthy lung tissues of CHI3L1 WT and CHI3L1 KO mice (Supplementary Figure S6B). The level of SOD1 protein was also significantly increased in lung tissues from metastatic and tumor sites of CHI3L1 KO mice, and the immune histochemical staining result confirmed this (Figure 6D and Supplementary Figure S6C and S6D). To determine whether increased SOD1 expression caused by the depletion of CHI3L1 induces ER stress, ER chaperone protein levels were observed in CHI3L1 and SOD1-depleted lung cancer cells. The depletion of CHI3L1 increased SOD1 expression and ER chaperone protein levels. However, in the case of the double depletion of CHI3L1 and SOD1, the ER chaperone protein levels were similar to that of the control group (Figure 6E). In addition, PERK protein levels were increased by depletion of CHI3L1 in lung cancer cells (Figure 6F). Double depletion of CHI3L1 and SOD1 reduced the PERK protein levels compared with the depletion of CHI3L1 cancer cells (Figure 6F). We investigated the expression of ER stress-mediated apoptotic marker proteins. The double depletion of CHI3L1 and SOD1 suppressed the expression of CHOP and cleaved caspase-12 that was induced by the depletion of CHI3L1 (Figure 6G). In addition, we performed the TUNEL assay to detect the apoptotic cells. The depletion of CHI3L1 increased the proportion of apoptotic cancer cells (Figure 6H). However, the double depletion of CHI3L1 and SOD1 reduced the number of apoptotic cells (Figure 6H). These results indicated that the depletion of CHI3L1 upregulated SOD1 expression, which induces ER stress responses and apoptosis in lung cancer cells.

### CHI3L1 KO suppresses lung cancer metastasis in mice through SOD1 upregulation

**Figure 7:**
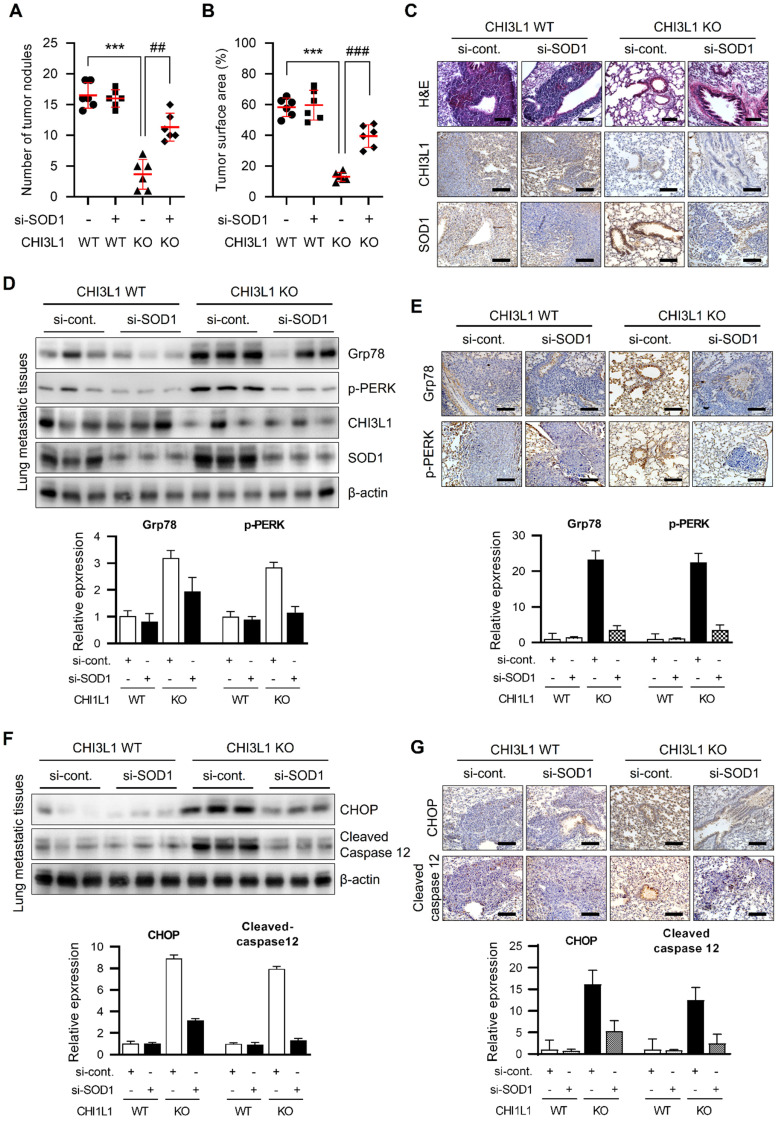
** CHI3L1 KO mice suppress lung metastasis through SOD1 expression.** (A-G) A549 cells (1X10^7^ cells) were injected intravenously scramble si-RNA or si-SOD1 were injected intravenously thrice a week for seven weeks. n=6 per group. (**A**) The number of metastatic nodules on the lung surface was counted and quantified (n=6). (**B**) The tumor surface areas on the lung tissue were measured from H&E staining images and quantified as a percentage of the total lung surface area (n=6). (**C**) Representative H&E staining images of metastatic lung tissues excised from each group. H&E staining were repeated from three independent experiments. (**D**) The lung tissue extracts were subjected to immunoblot analysis with indicated antibodies. The positive cells in immunohistochemistry staining was measured and calculated. Data are presented as mean ± standard deviation (SD) from two independent experiments. (**E**) Representative immunohistochemical images of lung tissues using indicated antibodies in each group. Immunohistochemical staining were repeated from three independent experiments. The positive cells in immunohistochemistry staining was measured and calculated. Data are presented as mean ± standard deviation (SD) from two independent experiments. (**F**) The lung tissue extracts were subjected to immunoblot analysis with indicated antibodies. The intensity of each band was measured and the ratio of the amount of each protein to β-actin was calculated. Data are presented as mean ± standard deviation (SD) from two independent experiments. (**G**) Representative immunohistochemical images of lung tissues using indicated antibodies in each group. Immunohistochemical staining were repeated from three independent experiments.

To further investigate the role of SOD1 in *in vivo* metastasis in CHI3L1 WT and KO mice, A549 cells were injected into tail veins and control siRNA and SOD1 siRNA were intravenously injected into CHI3L1 WT and KO mice a three times per week for 7 weeks. The CHI3L1 KO group had significantly fewer metastatic nodes in lung tissues compared with the CHI3L1 WT group (Figure 7A). The SOD1 siRNA-injected CHI3L1 WT group did not change the number of metastatic nodes in lung tissues compared with the control siRNA-injected CHI3L1 WT group. However, SOD1 siRNA-injected CHI3L1 KO mice increased the number of metastatic nodes in lung tissues compared to the control siRNA-injected CHI3L1 KO group (Figure 7A). The surface areas of metastatic lung tissues with the CHI3L1 KO group was lower than that of the CHI3L1 WT group (Figure 7B). Consistent with the decrease in the number of metastatic nodes, surface areas of metastatic lung were significantly reduced in the control siRNA-injected CHI3L1 KO group compared with the other groups (Figure 7B). H&E staining were performed on metastatic lung tumors (Figure 7C).

To determine whether SOD1 affects ER stress in CHI3L1 KO mice, UPR-associated protein levels were observed in metastatic lung tissues. Grp78 and p-PERK expression was increased in CHI3L1 KO mice metastatic lung tissues. However, in the SOD1 siRNA-injected CHI3L1 KO group, UPR-associated protein levels were increased compared with the control siRNA-injected CHI3L1 KO mice (Figure 7D). Furthermore, the immunohistochemical results showed that the expression of Grp78 and phosphorylated PERK was increased in metastatic lung tissues of control siRNA-injected CHI3L1 KO mice (Figure 7E). Then the SOD1 siRNA-injected CHI3L1 KO increased the UPR-associated protein levels compared with control siRNA-injected CHI3L1 KO mice metastatic lung tissue (Figure 7E). We investigated the expression of ER stress-mediated apoptotic marker proteins. As a result, SOD1 siRNA-injected CHI3L1 KO mice suppressed the expression of CHOP and cleaved caspase-12 induced in the control siRNA-injected CHI3L1 KO mice group (Figure 7F). Consistent with immunoblotting results, immunohistochemistry analysis also showed decreased CHOP and cleaved caspase 12 levels in lung metastatic tissues of SOD1 siRNA-injected CHI3L1 KO mice (Figure 7G). These results indicated that CHI3L1 deficiency increased ER stress-mediated apoptotic cell death through SOD1 expression, thus inhibiting lung metastasis.

## Discussion

CHI3L1 is known to be important in various diseases such cancers, allergic diseases, and inflammatory diseases 19-21. We have reported the importance of CHI3L1 in tumor development, especially lung cancer 22-27. As it is important to understand the precise biological function of CHI3L1 in lung cancer, we investigated the potential novel pathophysiological function of CHI3L1 in lung tumorigenesis. We analyzed the changes in the protein distribution in CHI3L1-overexpressing cells using LC-MS/MS analysis, and found 451 DEPs compared with the control. The biological function of the 451 DEPs was analyzed and it was found that the proteins with ER-associated function were prevalent in CHI3L1-overexpressing ells. In this study, this was confirmed using fluorescence staining and fractionation, which showed that CHI3L1 was co-localized with the ER marker protein Grp78 in both normal and cancerous cells. It was also found that CHI3L1 and Grp78 interact higher in lung cancer cells than normal lung cells.

The ER is mainly involved in protein folding; in cancer, misfolded proteins accumulated and consequently ER stress occurs 5, 6. When ER stress occurs, the levels of ER chaperones such as Grp78, PDI, and CNX are increased to improve the folding ability of ER. In this study, we compared the effect of CHI3L1 on the ER chaperone levels in normal cells and cancer cells. In normal cells, CHI3L1 did not affect ER chaperone proteins (Grp78 and PDI). However, under ER stress conditions, the depletion of CHI3L1 increased ER chaperone protein levels in normal lung cells. Unlike in normal cells, the depletion of CHI3L1 increased ER chaperone protein levels in lung cancer cells. In addition, the depletion of CHI3L1 further increased ER chaperone protein levels under ER stress conditions.

The accumulation of abnormal proteins in the ER by various factors disrupts the ER functions. Cells try to solve this problem by activating the UPR, the ER stress pathway. The UPR process is mediated by the IRE1, ATF and PERK proteins. In the absence of stress in normal cells, these three proteins bind to Grp78 and exist in an inactive state 7-10. However, when stress increases, Grp78 is isolated from IRE1, ATF6, and PERK proteins and activates the UPR 9, 57, 58. The function of the UPR can be classified into; 1) gene expression related to ER function through transcription increase (IRE1a); 2) interruption of new protein synthesis through translation inhibition (PERK); 3) degradation of unfolded proteins through ERAD (ATF6). Three stress sensor proteins regulate the expression of various genes related to protein homeostasis regulation through the activation of each signaling pathway and downstream transcription factors, thereby exhibiting a response to ER 11-18. The results of our study show that CHI3L1 depletion did not respond to ER stress, which indicated that stress sensor protein (IREα1, PERK, and ATF6) expression in normal cells was unaffected. This means that UPR is not activated by CHI3L1 in normal cells. However, the depletion of CHI3L1 increased the phosphorylation of PERK in cancer cells. It can be assumed that the response of CHI3L1 to the ER is different in normal cells and cancer cells. As cancer cells have specific a biological response to the surrounding microenvironment, the UPR response to it appears different from that of normal cells. However, when stress is induced by thapsigargin treatment, ER stress is induced in both normal cells and cancer cells. When cells are stressed, they actively respond to cells that produce various proteins. Our data show that under ER stress conditions, the depletion of CHI3L1 greatly induced further ER stress and eventually activated the UPR in both normal and cancerous lung cells, but to a greater extent in lung cancer cells.

PERK is a major protein responsible for reducing mRNA translation owing to ER stress, preventing newly synthesized proteins from entering the already stressed ER compartment. The translational attenuation is regulated by the phosphorylation of eIF2α. The phosphorylation of eIF2α suppresses the recycling of eIF2α in the form of the GTP-bound active, which is required for the initiation step in polypeptide chain synthesis. This also enables the preferential translation of UPR-dependent genes such as ATF4. We confirmed that the depletion of CHI3L1 induced the phosphorylation of PERK and increased the eIF2α and ATF4 levels in cancer cells, but not normal cells. Under stress conditions, the deficiency of CHI3L1 increased the PERK protein levels in both normal cells and cancer cells, but to a greater extent in cancer cells. ER stress is activated as a cell defense mechanism against various stresses, but it induces apoptosis when ER stress is continuously generated or misfolded proteins are excessively accumulated. Phosphorylated eIF2α induces the expression of the ATF protein, which induces the CHOP protein, blocking the expression of Bcl2. Bcl2 is an anti-apoptotic protein; the inhibition of Bcl2 expression eventually leads to apoptosis 41, 44-46. In addition, caspase-12 activation by ER stress moves it from the ER to the cytosol, which in turn activates effector caspases, resulting in apoptosis 44, 52, 53. We confirmed that the depletion of CHI3L1 did not cause ER stress-mediated apoptosis in normal cells. However, it was found that the depletion of CHI3L1 increased CHOP and cleaved caspase-12 levels in cancer cells. Similarly, it was confirmed that ER stress-mediated apoptotic marker protein levels were increased in tumor tissue and lung metastatic tissues of CHI3L1 KO mice. We also confirmed that Grp78 and PERK were overexpressed in lung cancer tissues in patients with NSCLS. Increasing the expression of Grp78 and p-PERK is one of the important strategies for inducing cancer cell death. While Grp78 is an essential protein for cancer cell survival and proliferation, its increased expression can induce cancer cell death. Additionally, p-PERK regulates enzyme activity in cells under conditions of imbalanced stress, determining cell survival. Therefore, increasing the expression of Grp78 and p-PERK can trigger stress responses in cancer cells, leading to their death. Various methods are being developed to increase the expression of Grp78 and p-PERK, which are considered as new strategies for anticancer therapy. Based on our results, depletion of CHI3L1 in cancer cells activates UPR and induces stress response, providing a new strategy for anti-cancer therapy. These results indicate that the depletion of CHI3L1 induced ER stress-mediated apoptosis in cells, resulting in the suppression of lung tumor growth and metastasis.

However, the exact mechanism of how CHI3L1 causes ER stress and thus induces apoptosis and inhibits tumorigenesis is unknown. From the GENEMANIA database, we found that the SOD1 protein forms an interaction network between CHI3L1 and ER stress. In addition, we confirmed that CHI3L1 physically interacts with SOD1. Several papers reported that SOD1 accumulation activates the ER stress-mediated UPR pathway 54-56, 59. The accumulation of SOD1 has been considered as an ER stress marker. SOD1 colocalizes with ER chaperone proteins, including Grp78. Moreover, it has been reported that the ER chaperone protein PDI is upregulated in cells containing an accumulation of SOD1 59-61. Our results showed that the depletion of CHI3L1 increased SOD1 expression in cancer cells but not in normal cells. Furthermore, the depletion of CHI3L1 significantly increased SOD1 expression in *in vivo* tumor and lung tissues. Several groups have identified SOD1 as a potential therapeutic target in cancer. SOD1 associates cell survival and tumor growth in the tumor microenvironment ^63^. The double depletion of CHI3L1 and SOD1 decreased the ER chaperone proteins (Grp78 and PDI) and PERK signaling proteins compared with the depletion of CHI3L1 in lung cancer cells and metastatic lung tumor tissues. In addition, ER stress-mediated apoptotic marker protein levels also much greatly decreased in the dual depletion of CHI3L1 and SOD1 in *in vitro* and *in vivo*. Here, we showed that the depletion of CHI3L1 induces ER stress-mediated lung cancer cell death through SOD1 upregulation.

The present study provides important insights into the role of CHI3L1 in the regulation of ER stress associated UPR function and apoptosis, as well as the SOD1 dependent regulation of tumor growth.

## Supplementary Material

Supplementary figures and table.
