# Induction of ER stress-mediated apoptosis through SOD1 upregulation by deficiency of CHI3L1 inhibits lung metastasis: Erratum

**DOI:** 10.7150/thno.98971

**Published:** 2024-10-04

**Authors:** Ji Eun Yu, In Jun Yeo, Seung Sik Yoo, Sung-hyun Kim, Dong Ju Son, Jaesuk Yun, Sang-Bae Han, Jin Tae Hong

**Affiliations:** College of Pharmacy and Medical Research Center, Chungbuk National University, 194-31, Osongsaengmyeong 1-ro, Osong-eup, Cheongju-si, Chungbuk 28160, Republic of Korea.

The authors would like to issue a correction regarding the figures in our published paper. We have identified an error where the CHI3L1 blot is duplicated in Figures 3A and 3D. Accordingly, revise the CHI3L1 blot in Figure 3D. This mistake occurred during the final preparation of the manuscript and figures.

Additionally, we have discovered that the images for CHI3L1 KO lung metastatic tissue in Figures 5F and 6D appear similar. To avoid confusion, we have corrected the representative image for CHI3L1 KO lung metastatic tissue in Figure 5F.

We confirm that these corrections do not affect the original data or the conclusions of the paper. The data in both the incorrect and correct figures are based on the same analysis, and there is no change to the results or interpretations presented in the study.

We apologize for any inconvenience caused by these errors and appreciate your understanding.

## Figures and Tables

**Figure 3:**
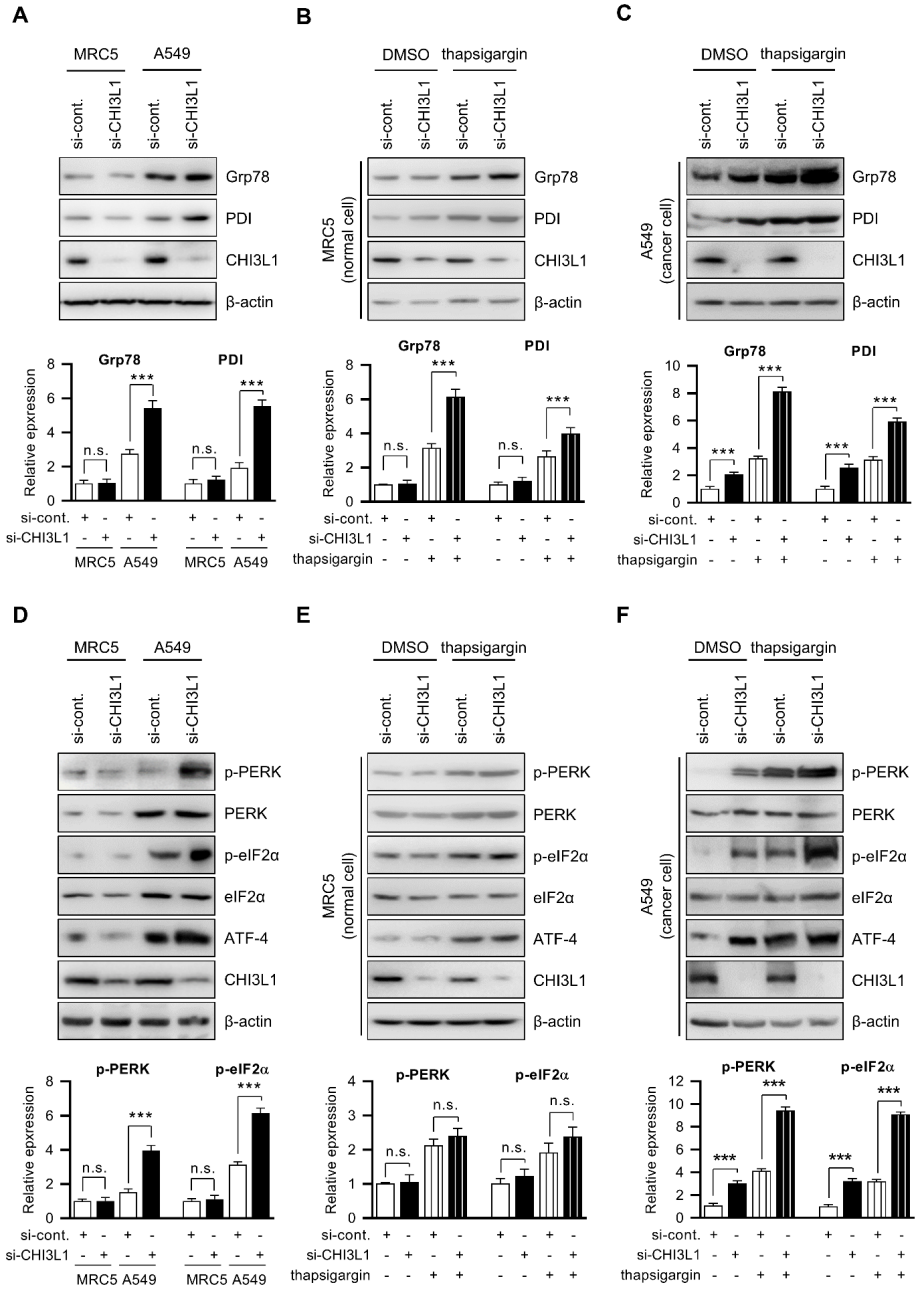
** Depletion of CHI3L1 activates ER stress and induces PERK and eIF2α in A549 cells.** (**A**) MRC5 (normal lung cell) and A549 (lung cancer cell) cells were transfected with control siRNA (si-cont.) or CHI3L1 siRNA (si-CHI3L1). The cell lysates were subjected to immunoblot analysis with the indicated antibodies. The intensity of each band was measured and the ratio of the amount of each protein to β-actin was calculated. Data are presented as mean ± standard deviation (SD) from two independent experiments. ***, *P* < 0.001; n.s., not significant. (**B**) MRC5 cells were transfected with si-control or si-CHI3L1, then treated with 1 uM thapsigargin (ER stress inducer) for 18 h. The cell lysates were subjected to immunoblot analysis with the indicated antibodies. The intensity of each band was measured and the ratio of the amount of each protein to β-actin was calculated. Data are presented as mean ± standard deviation (SD) from two independent experiments. ***, *P* < 0.001; n.s., not significant. (**C**) A549 cells were transfected with si-control or si-CHI3L1, then treated with 1 uM thapsigargin for 18 h. The cell lysates were subjected to immunoblot analysis with the indicated antibodies. The intensity of each band was measured and the ratio of the amount of each protein to β-actin was calculated. Data are presented as mean ± standard deviation (SD) from two independent experiments. ***, *P* < 0.001. (**D**) MRC5 (normal lung cell) and A549 (lung cancer cell) cells were transfected with control siRNA (si-cont.) or CHI3L1 siRNA (si-CHI3L1). The cell lysates were subjected to immunoblot analysis with the indicated antibodies. The intensity of each band was measured and the ratio of the amount of each protein to β-actin was calculated. Data are presented as mean ± standard deviation (SD) from two independent experiments. n.s., not significant. (**E**) MRC5 cells were transfected with si-control or si-CHI3L1, then treated with 1 uM thapsigargin (ER stress inducer) for 18 h. The cell lysates were subjected to immunoblot analysis with the indicated antibodies. (**F**) A549 cells were transfected with si-control or si-CHI3L1, then treated with 1 uM thapsigargin for 18 h. The cell lysates were subjected to immunoblot analysis with the indicated antibodies. The intensity of each band was measured and the ratio of the amount of each protein to β-actin was calculated. Data are presented as mean ± standard deviation (SD) from two independent experiments. ***, *P* < 0.001.

**Figure 5:**
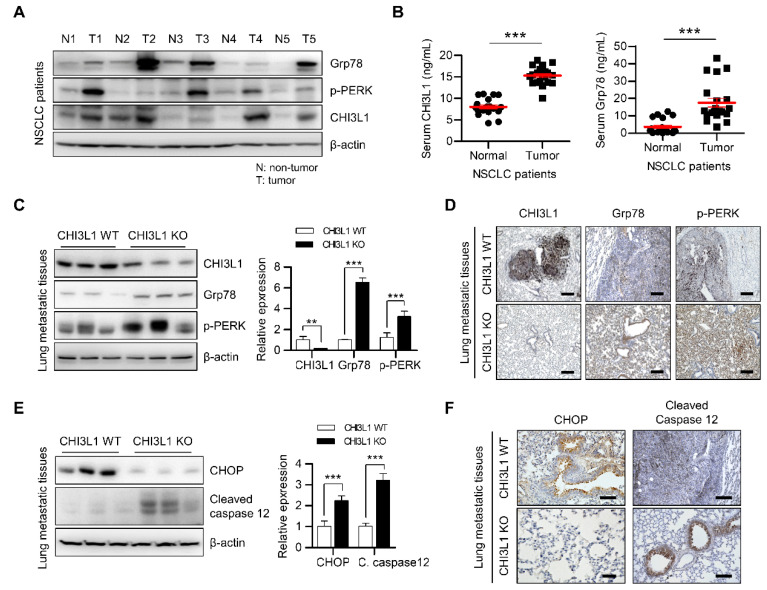
** The metastatic lung tissues of CHI3L1 KO mice induces ER stress mediated apoptosis.** (**A**) The lung tissue extracts of NSCLC patients were subjected to immunoblot analysis with indicated antibodies. (**B**) The serum of NSCLC patients was subjected to ELISA analysis with indicated proteins. (**C**) The metastatic lung tissues extract of mice were subjected to immunoblot analysis with indicated antibodies. The intensity of each band was measured and the ratio of the amount of each protein to β-actin was calculated. Data are presented as mean ± standard deviation (SD) from two independent experiments. ***, *P* < 0.001. (**D**) Representative immunohistochemical images of metastatic lung tissues in mice using anti-CHI3L1, anti-Grp78, and anti-p-PERK antibodies in each group. Scale bar, 100 μm. (**E**) The metastatic lung tissues extracts of mice were subjected to immunoblot analysis with indicated antibodies. The intensity of each band was measured and the ratio of the amount of each protein to β-actin was calculated. Data are presented as mean ± SD from two independent experiments. ***, *P* < 0.001. (**F**) Representative immunohistochemical images of metastatic lung tissues in mice using anti-CHOP and anti-cleaved caspase12 antibodies in each group. Scale bar, 100 μm.

