# Supplementary material for: Induction of ER stress-mediated apoptosis through SOD1 upregulation by deficiency of CHI3L1 inhibits lung metastasis
Source: Theranostics. 2023 Apr 29;13(8):2693–709. doi: 10.7150/thno.82898 (PMC10196820; doi:10.7150/thno.82898)
Supplement: Supplementary file 1 — Supplementary figures and table. [file thnov13p2693s1.pdf]

Supplementary Figure Legends

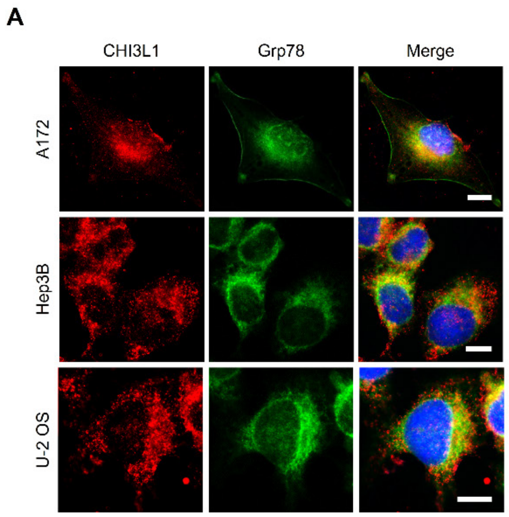

Supplementary Figure S1. CHI3L1 co-localizes with Grp78 at the ER.

(**A**) The 4% paraformaldehyde fixed A172 (glioblastoma cell), Hep3B (hepatoma cell), and U-2 OS (osteosarcoma cell) cells were co-stained with anti-CHI3L1 and anit-Grp78. Scale bar, 10 μm.

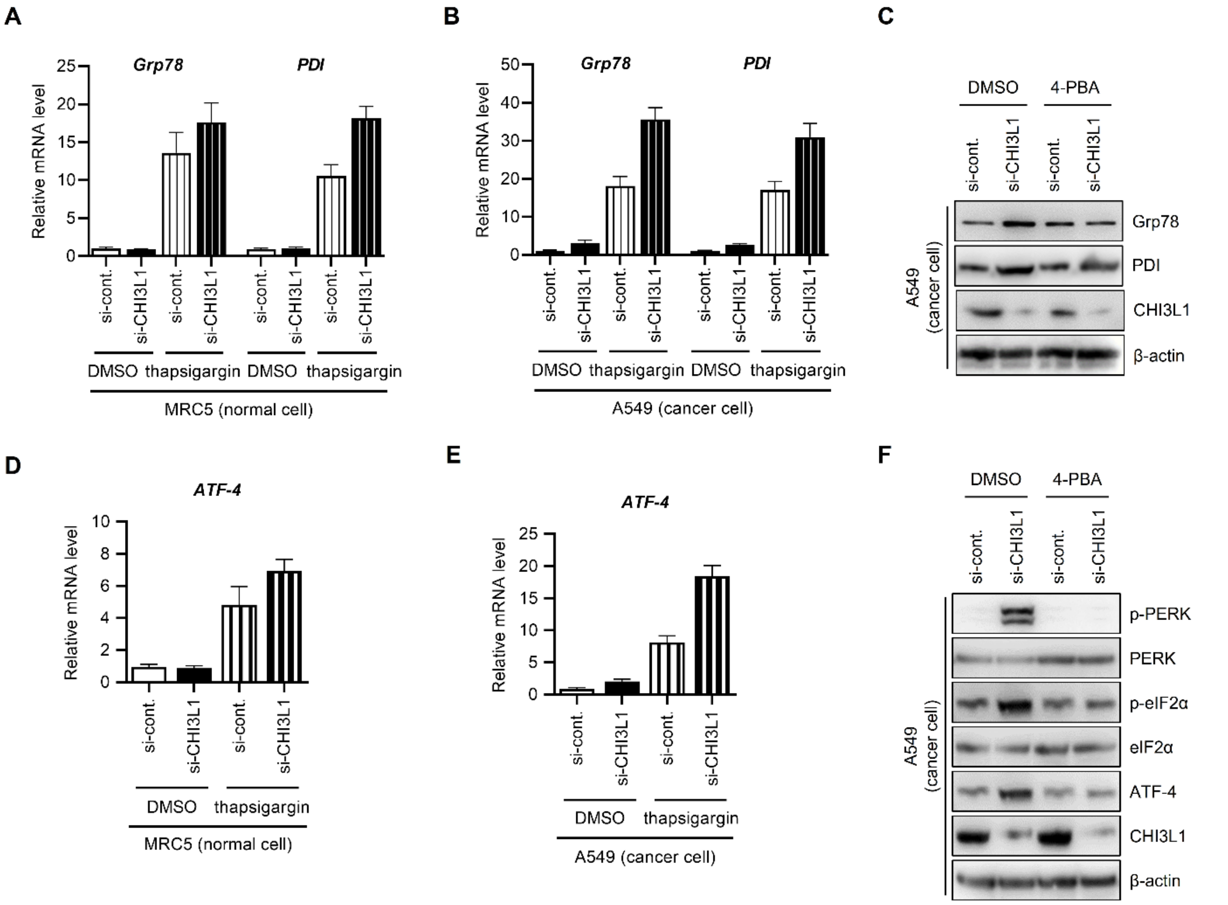

Supplementary Figure S2. Depletion of CHI3L1 induces ER stress in lung cancer cells.

(**A**) MRC5 cells were transfected with si-control or si-CHI3L1, then treated with 1 uM thapsigargin (ER stress inducer) for 18 h. The relative mRNA levels were quantified using RT-qPCR. The values are normalized to *GAPDH* mRNA levels. Data are presented as mean ± standard deviation (SD) from three independent experiments. (**B**) A549 cells were transfected with si-control or si-CHI3L1, then treated with 1 uM thapsigargin for 18 h. The relative mRNA levels were quantified using RT-qPCR. The values are normalized to *GAPDH* mRNA levels. Data are presented as mean ± standard deviation (SD) from three independent experiments. (**C**) A549 cells were transfected with si-control or si-CHI3L1, then treated with 1 mM 4-PBA (ER stress inhibitor) for 18 h. The cell lysates were subjected to immunoblot analysis with the indicated antibodies. (**D**) MRC5 cells were transfected with si-control or si-CHI3L1, then treated with 1 uM thapsigargin (ER stress inducer) for 18 h. The relative mRNA levels were quantified using RT-qPCR. The values are normalized to *GAPDH* mRNA levels. Data are presented as mean ± standard deviation (SD) from three independent experiments. (**E**)

A549 cells were transfected with si-control or si-CHI3L1, then treated with 1 uM thapsigargin for 18 h. The relative mRNA levels were quantified using RT-qPCR. The values are normalized to *GAPDH* mRNA levels. Data are presented as mean ± standard deviation (SD) from three independent experiments. (**F**) A549 cells were transfected with si-control or si-CHI3L1, then treated with 1 mM 4-PBA (ER stress inhibitor) for 18 h. The cell lysates were subjected to immunoblot analysis with the indicated antibodies.

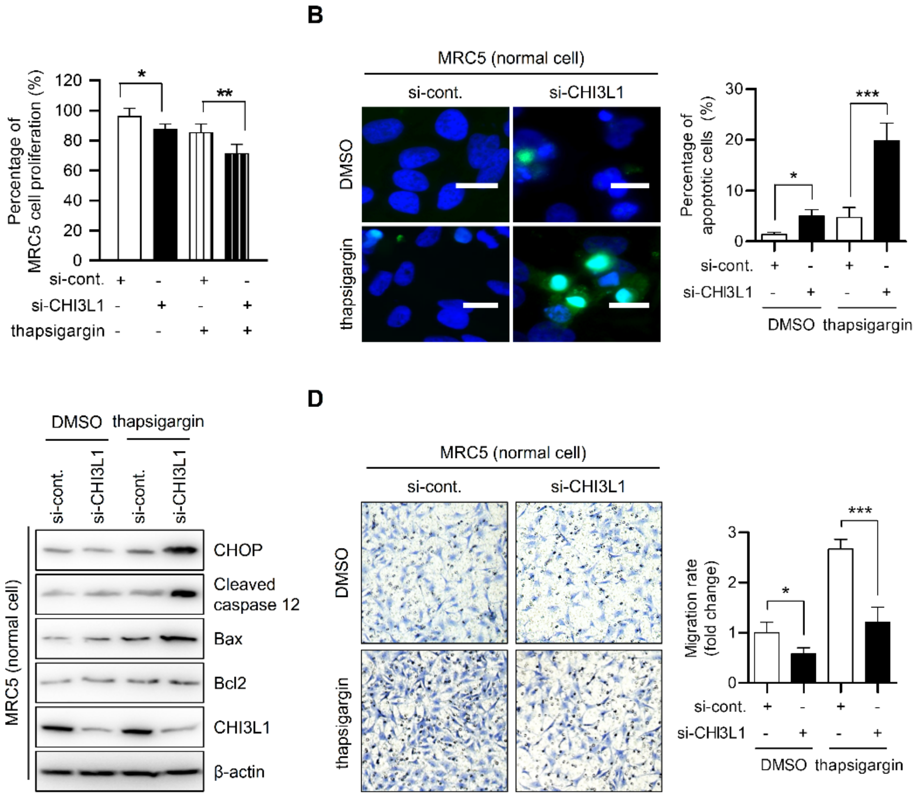

Supplementary Figure S3. Depletion of CHI3L1 under ER stress induces further ER stress-mediated apoptosis in normal cells.

(**A**) MRC5 cells were transfected with si-control or si-CHI3L1, then treated with 1 uM thapsigargin for 18 h. The cell viability was measured using the MTT assay. The data are presented as the mean ± SD of three independent experiments. *, *P* < 0.05; **, *P* < 0.01. (**B**) Representative fluorescence microscopic images showing DAPI (blue) and TUNEL (green) staining in MRC5 cells transfected with si-control or si-CHI3L1 in the absence or presence of the thapsigargin (1 μM). Scale bar, 20 μm. The number of positively stained cells was counted in three different fields and averaged. The data are presented as the mean ± SD of three independent experiments. *, *P* < 0.05; ***, *P* < 0.001. (**C**) MRC5 cells were transfected with si-control or si-CHI3L1, then treated with 1 uM thapsigargin for 18 h. The cell lysates were subjected to immunoblot analysis with the indicated antibodies. (**D**) si-control or si-CHI3L1 transfected MRC5 cells were seeded in the upper chamber and incubated at 37 °C for 18 h. The migrated cells on the bottom chamber were stained with 0.1% crystal violet. Data are presented as mean ±SD from three independent experiments. The number of migrated cells was counted in three different fields and averaged. The data are presented as the mean ± SD of three independent experiments. *, *P* < 0.05; ***, *P* < 0.05.

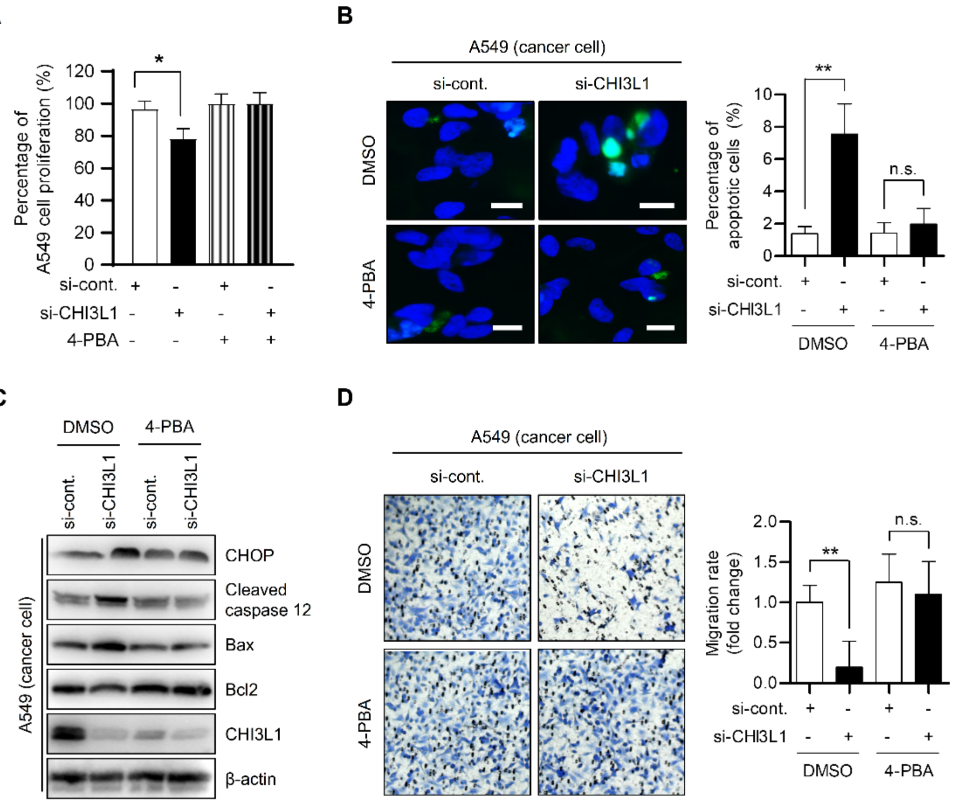

Supplementary Figure S4. CHI3L1 induces ER stress in lung cancer cells.

(**A**) A549 cells were transfected with si-control or si-CHI3L1, then treated with 1 mM 4-PBA for 18 h. The cell viability was measured using the MTT assay. The data are presented as the mean ± SD of three independent experiments. *, $P < 0.05$. (**B**) Representative fluorescence microscopic images showing DAPI (blue) and TUNEL (green) staining in A549 cells transfected with si-control or si-CHI3L1 in the absence or presence of the 4-PBA (1 mM). Scale bar, 10 μm. The number of positively stained cells was counted in three different fields and averaged. The data are presented as the mean ± SD of three independent experiments. **, $P < 0.01$. (**C**) A549 cells were transfected with si-control or si-CHI3L1, then treated with 1 mM 4-PBA for 18 h. The cell lysates were subjected to immunoblot analysis with the indicated antibodies. (**D**) si-control or si-CHI3L1 transfected A549 cells were seeded in the upper chamber and incubated at 37 °C for 18 h. The migrated cells on the bottom chamber were stained with 0.1% crystal violet. Data are presented as mean ±SD from three independent experiments. The number of migrated cells was counted in three different fields and averaged. The data are presented as the mean ± SD of three independent experiments. **, $P < 0.01$.

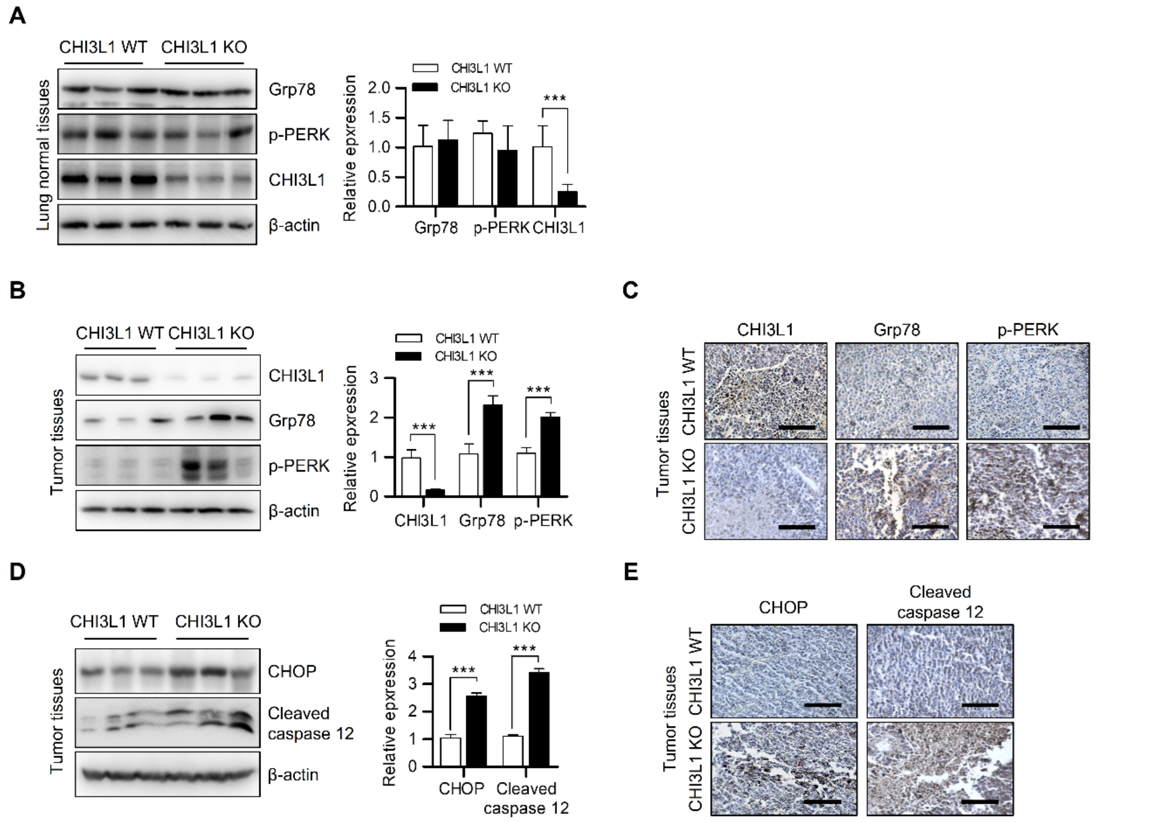

Supplementary Figure S5. The tumor tissues of CHI3L1 KO mice induce ER stress mediated apoptosis.

(**A**) The lung tissue extracts were subjected to immunoblot analysis with indicated antibodies. The intensity of each band was measured and the ratio of the amount of each protein to β-actin was calculated. Data are presented as mean ± standard deviation (SD) from two independent experiments. ***, $P < 0.001$. (**B**) The tumor tissue extracts were subjected to immunoblot analysis with indicated antibodies. The intensity of each band was measured and the ratio of the amount of each protein to β-actin was calculated. Data are presented as mean ± standard deviation (SD) from two independent experiments. ***, $P < 0.001$. (**C**) Representative immunohistochemical images of tumor tissues using anti-CHI3L1, anti-Grp78, and anti-p-PERK antibodies in each group. Scale bar, 100 μm. (**D**) The metastatic lung tissues extracts were subjected to immunoblot analysis with indicated antibodies. The intensity of each band was measured and the ratio of the amount of each protein to β-actin was calculated. Data are presented as mean ± standard deviation (SD) from two independent experiments. ***, $P < 0.001$. (**E**) Representative immunohistochemical images of tumor tissues using anti-CHOP, anti-cleaved caspase 12 antibodies in each group. Scale bar, 100 μm.

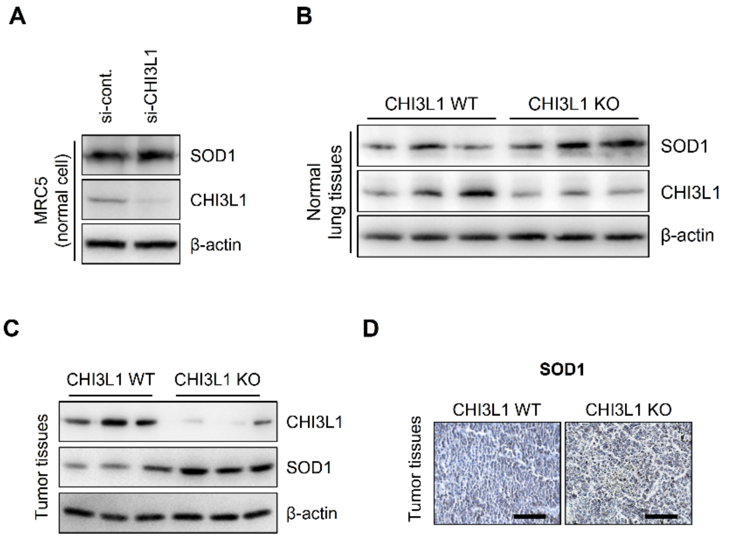

Supplementary Figure S6. The expression of SOD1 increases in the tumor tissues of CHI3L1 KO mice.

(**A**) MRC5 cells were transfected with control siRNA or si-CHI3L1.The cell lysates were subjected to immunoblot analysis with the indicated antibodies (**B**) The lung tissue extracts were subjected to immunoblot analysis with indicated antibodies. (**C**) The tumor tissue extracts were subjected to immunoblot analysis with indicated antibodies. (**D**) Representative immunohistochemical images of tumor tissues using anti-SOD1 antibodies in each group. Scale bar, 100 μm.

Supplementary Table 1. List of differentially expressed proteins between CHI3L1-expressing cell and vector-expressing cell

Symbol	logFC	adj-P-Val	Symbol	logFC	adj-P-Val
VCL	-4.319	0.028	SOD2	8.087	0.000
GMFG	-3.905	0.004	ICAM1	4.556	0.020
TMSB10	-3.497	0.017	IL1B	4.550	0.001
NAPRT	-3.473	0.033	MARCKS	4.268	0.007
STK10 ENSG00000072786	-3.124	0.044	PLG	3.930	0.006
PRDX2	-3.072	0.028	GDF15	3.262	0.020
TPT1	-2.949	0.028	NPTN	2.928	0.046
NASP	-2.785	0.028	GOT2	2.899	0.039
CORO1A	-2.734	0.017	TAP2	2.773	0.020
ITGB5	-2.588	0.037	MMP9	2.755	0.020
NUDC	-2.587	0.020	VTN	2.703	0.038
NRGN	-2.469	0.020	TIMP1	2.644	0.017
PXN	-2.418	0.049	ALCAM	2.551	0.017
TPM4	-2.290	0.020	CORO7-PAM16	2.507	0.028
S100A4	-2.285	0.017	ATP2B1	2.474	0.020
SNX3	-2.257	0.044	MTHFD2	2.283	0.020
COPB1	-2.243	0.040	GPD2	2.210	0.020
YBX1	-2.215	0.028	TMEM43	2.175	0.028
TMSB4X	-2.207	0.034	DHRS9	2.118	0.049
SERBP1	-2.203	0.042	HBD	2.101	0.020
CLPX	-2.199	0.028	PLAUR	2.059	0.028
S100A6	-2.167	0.035	TAP1	2.053	0.039
HMGB1	-2.158	0.028	PPIF ENSG00000108179	2.030	0.020
PNP	-2.122	0.020	SAFB	1.783	0.037
CAPG	-2.121	0.020	PMPCA ENSG00000165688	1.753	0.038
HSP70	-2.055	0.021	DDX21	1.722	0.031
NA	-1.983	0.020	ACADVL	1.677	0.038
MYO1E	-1.983	0.039	SQOR	1.563	0.028
YWHAB	-1.977	0.038	CD44	1.551	0.028
COTL1	-1.974	0.029	ALDH2	1.543	0.048
LDHB	-1.879	0.024	ADGRE5	1.474	0.048
ST13	-1.823	0.024	CAMK1	1.473	0.033
HSPA4	-1.816	0.025	NES	1.449	0.039
SH3BGRL3	-1.806	0.021	DLD	1.394	0.049
UBE2V2	-1.771	0.025	COX7C	1.370	0.039
EZR	-1.770	0.049	GPC4	1.278	0.046
CKAP4	-1.759	0.050	TOMM70	1.236	0.038
RPL13	-1.755	0.049	RAB1A	1.218	0.049
FSCN1	-1.751	0.028	ACO2	1.215	0.043
SRSF2	-1.730	0.039	SHMT2	1.195	0.046
PRKCSH	-1.729	0.028	HADHA	1.103	0.049
PSMC1	-1.718	0.028	HSPA9	1.096	0.049
ATIC	-1.712	0.049	PHB	1.081	0.050
GSN	-1.692	0.021	UQCRC1	1.080	0.049
FABP5	-1.613	0.027	FH	1.071	0.046
TUBA1A	-1.600	0.042	UQCRC2	1.051	0.049
PGAM1 ENSG00000171314	-1.597	0.023	MATR3	1.027	0.049
DPYSL2	-1.596	0.020	HNRNPA1	0.905	0.049
WDR1	-1.589	0.028	RAB21	0.855	0.049
HSP90AA1	-1.581	0.024	CYC1	0.841	0.049
HDGF	-1.548	0.042			
EEF1G	-1.545	0.049			

GAA	-1.525	0.049
ACTN4	-1.525	0.025
EIF4G1	-1.523	0.039
GLO1	-1.510	0.025
CAP1 ENSG00000131236	-1.505	0.028
ACTR3	-1.496	0.031
PRDX6	-1.492	0.043
GDI2	-1.480	0.046
ACTR2	-1.461	0.049
RNH1	-1.428	0.031
UBA1	-1.425	0.040
SAMHD1	-1.421	0.031
PSMA6	-1.419	0.037
TAGLN2	-1.413	0.033
CLTA	-1.413	0.049
ARHGDIB	-1.410	0.028
RPS11	-1.362	0.039
YWHAQ	-1.352	0.046
CFL1	-1.345	0.028
GSTP1	-1.342	0.038
PFN1	-1.330	0.031
ARPC3 ENSG00000111229	-1.327	0.043
SEPTIN2	-1.327	0.035
PARK7	-1.291	0.040
PPP2R1A	-1.253	0.050
PTGES3	-1.247	0.049
TALDO1	-1.239	0.039
CORO1C	-1.232	0.049
ALDOA	-1.194	0.038
MSN	-1.191	0.038
NPC2	-1.186	0.031
EEF1D	-1.169	0.034
TKT	-1.165	0.049
CLIC1	-1.146	0.030
CA2	-1.143	0.049
RPS20	-1.118	0.046
ARPC2	-1.095	0.049
TPM3	-1.084	0.041
CTPS1	-1.083	0.044
DOCK10	-1.073	0.044
LDHA	-1.050	0.050
HSPB1	-1.019	0.049
RPS12	-0.970	0.039
MYL6	-0.965	0.039
ENO1 ENSG00000074800	-0.952	0.040
PPIA	-0.949	0.049
TLN1	-0.937	0.039
ACTB	-0.925	0.040
CCT6A	-0.919	0.044
PKM	-0.905	0.049
GAPDH	-0.851	0.044
RBM3	-0.838	0.039
PGK1	-0.830	0.049
RPS7	-0.770	0.049
CCT8	-0.768	0.049
ACTN1	-0.746	0.049
LGALS1	-0.674	0.049
